# Activation of thousands of genes in the lungs and kidneys by sepsis is countered by the selective nuclear blockade

**DOI:** 10.3389/fimmu.2023.1221102

**Published:** 2023-08-11

**Authors:** Huan Qiao, Jozef Zienkiewicz, Yan Liu, Jacek Hawiger

**Affiliations:** ^1^ Department of Medicine, Division of Allergy, Pulmonary and Critical Care Medicine, Vanderbilt University School of Medicine, Nashville, Tennessee, TN, United States; ^2^ Department of Veterans Affairs, Tennessee Valley Health Care System, Nashville, Tennessee, TN, United States; ^3^ Department of Molecular Physiology and Biophysics, Vanderbilt University School of Medicine, Nashville, Tennessee, TN, United States

**Keywords:** polymicrobial sepsis, nuclear transport checkpoint inhibitor (NTCI), cell-penetrating peptides (CPP), next generation sequence (NGS), inflammation, cecal microbiome, acute respiratory distress syndrome (ARDS), acute kidney injury (AKI)

## Abstract

The steady rise of sepsis globally has reached almost 49 million cases in 2017, and 11 million sepsis-related deaths. The genomic response to sepsis comprising multi-system stage of raging microbial inflammation has been reported in the whole blood, while effective treatment is lacking besides anti-microbial therapy and supportive measures. Here we show that, astoundingly, 6,237 significantly expressed genes in sepsis are increased or decreased in the lungs, the site of acute respiratory distress syndrome (ARDS). Moreover, 5,483 significantly expressed genes in sepsis are increased or decreased in the kidneys, the site of acute injury (AKI). This massive genomic response to polymicrobial sepsis is countered by the selective nuclear blockade with the cell-penetrating Nuclear Transport Checkpoint Inhibitor (NTCI). It controlled 3,735 sepsis-induced genes in the lungs and 1,951 sepsis-induced genes in the kidneys. The NTCI also reduced without antimicrobial therapy the bacterial dissemination: 18-fold in the blood, 11-fold in the lungs, and 9-fold in the spleen. This enhancement of bacterial clearance was not significant in the kidneys. Cumulatively, identification of the sepsis-responsive host’s genes and their control by the selective nuclear blockade advances a better understanding of the multi-system mechanism of sepsis. Moreover, it spurs much-needed new diagnostic, therapeutic, and preventive approaches.

## 1 Introduction

Sepsis, a life-threatening complication of out-of-control infections, is rising globally (1). This overwhelming microbial inflammation encompassing multiple systems is caused by viruses (e.g., influenza, Ebola, Dengue, and SARS Coronavirus 2), Gram-positive and negative bacteria (e.g., Staphylococci, Streptococci, E. coli, Pseudomonas, and those causing wound infections in the battlefield), yeasts (e.g., Candida), and protozoa (e.g., Malaria). Severe infections evolving into sepsis, and then into septic shock, have become a pressing, worldwide public health problem that continues to grow in magnitude as the global population ages (1, 2). In the US, at least 1.7 million adults develop sepsis each year and 350,000 dies of the illness [CDC data (3)]. While one in three patients who died in the U.S. hospitals had sepsis, its mortality approaches that of heart attacks and exceeds the number of deaths from stroke (4). Neonates and the elderly constitute the most vulnerable to sepsis patients (5, 6), with a 28-days mortality rate exceeding 40% (7, 8). Thus, the prevention and very early control of severe viral, bacterial, fungal, and protozoal infections remains crucial for taming their progression into sepsis. Survivors suffer moderate to severe cognitive impairment as compared to other patients discharged from Intensive Care Units (8). In addition to the human cost of sepsis, its expenses rank highest among admissions for all diseases in the U.S. hospitals (9). They do not include expenses for the long-term care of survivors who suffer persistent developmental abnormalities (after neonatal sepsis) (2, 10) and incapacitating cognitive decline (after adult sepsis) (5). Thus, new approaches to the adjunctive therapy of sepsis are needed while sepsis-causing infections are increasingly difficult to control due to the growing multi-drug resistance (5).

The response to the sepsis-causing infections begins when the pattern-recognition receptors, Toll-like Receptors (TLRs), detect pathogen-associated molecular patterns, such as endotoxic lipopolysaccharide (LPS), the constituent of Gram-negative bacteria, the cause of the two-thirds of sepsis cases (11). Intracellular NOD-like receptor family senses conserved bacterial peptidoglycans (12). The innate immunity receptors, e.g., TLRs, are displayed in immune and non-immune cells, including microvascular endothelial cells and epithelial cells (13). Therein, they activate nuclear signaling pathways that mobilize families of the stress-responsive transcription factors, SRTFs (e.g., NF-κB, cFos, cJun, STAT1 and STAT3, NFAT, and Nrf2), and Metabolic Transcription Factors, MTFs (SREBPs and ChREBPs) (14–18). Their access to the inflammatory and metabolic regulomes in the cell’s nucleus is controlled by the nuclear transport checkpoint comprising Importin α5 and Importin β1, among others (19). Importin α5, aka karyopherin α1, is one of the six members of the importin α family (20). Importin α5 recognizes Nuclear Localization Sequence (NLS) on multiple proinflammatory SRTFs and metabolic transactivators, ChREBPs (21). Importin β1 selectively recognizes SREBPs (22). These proinflammatory and metabolic transcription factors activate a myriad of genes that encode mediators of microbial and metabolic inflammation (19).

Life-threatening sepsis progression is linked to the endothelial cells’ activation and subsequent injury in the small blood vessels (<100 μm diameter) supplying all organs (5). Microvascular endothelial activation and injury promote extravasation of leukocytes and lymphocytes and leakiness of small blood vessels. They contribute to the acute respiratory distress syndrome (ARDS) (23, 24) and the acute kidney injury (AKI) (25). These failing organs comprise the major therapeutic challenges in sepsis. In turn, Septic Shock, manifested by hypotension that is nonresponsive to fluid resuscitation or vasopressors, culminates sepsis (7). Until now, the genomic response to sepsis in the major organs other than blood has not been reported (26–28).

Herein, we submit that the expression of thousands of genes in the lungs (prone to ARDS) and kidneys (succumbing to AKI) was increased or decreased in experimental sepsis evolving from polymicrobial peritonitis. We also found that this genomic response to polymicrobial sepsis was countered by the selective nuclear blockade with the Nuclear Transport Checkpoint Inhibitor (NTCI), aka Nuclear Transport Modifier (NTM). Short (6 hours) treatment with the NTCI changed the expression of a multitude of organ-specific and shared genes in the lungs and kidneys responding to the experimental sepsis. Since humans and mice show on average 85% similarity of their DNA sequences that code for proteins (National Human Genome Research Institute) (29), our data provide relevant evidence that the major organs-based genomic response to sepsis is an important mechanism in its initiation, progression, and potential resolution.

The NTCIs are cell-penetrating anti-inflammatory and anti-metabolic peptides rapidly reaching immune cells in the blood and major organs e.g., lungs (15, 19). Moreover, they enter non-immune cells (e.g., endothelial cells lining blood vessels), as well as other cells (e.g., epithelial cells) in the major organs, attacked by sepsis-causing microbial agents (15, 18, 19). The NTCIs cross the cell membrane of immune and non-immune cells through the phospholipid bilayer without involving the chirally-specific receptor or transporter. Importantly, the NTCIs bypass the endosomal compartment thereby avoiding the potential intracellular degradation by lysosomal proteases (30). In the cytoplasm, the NTCIs simultaneously target the two nuclear import adaptor proteins, importin α5 and importin β1 (16, 19, 20) required for the nuclear translocation of proinflammatory SRTFs and MTFs (21). Thus, the NTCIs impede the nuclear entry of the key transcription factors, SRTFs and MTFs, thereby preventing their activation of the inflammatory and metabolic regulomes, respectively.

Astoundingly, we found that the selective nuclear blockade by NTCI resulted in reprogramming of 3,735 sepsis-induced genes in the lungs and 1,951 sepsis-induced genes in the kidneys. Moreover, the NTCI increased microbial clearance by reducing the sepsis-causing bacterial dissemination 18-fold in the blood, 9-fold in the spleen, and 11-fold in the lungs after the 12-hour treatment.

## 2 Materials and methods

### 2.1 Synthesis and purification of the cell-penetrating nuclear transport checkpoint inhibitor (NTCI)

The cell-penetrating NTCI peptide, cSN50.1 (AAVALLPAVLLALLAPCVQRKRQKLMPC, 2986 Da) and its FITC-labeled derivative (AAVALLPAVLLALLAGGK(FITC)GGPCVQRKRQKLMPC, 3,713 Da) were synthesized as described elsewhere (31). Briefly, the peptide chain was assembled through Solid Phase Peptide Synthesis (SPPS) according to standard Fmoc chemistry protocols using an automated peptide synthesizer FOCUS XC (AAPPTec, Louisville, KY). Crude peptides were removed from the resin with a TFA cleavage cocktail and purified by dialysis against double-distilled water in 1 kDa membrane (Spectra/Por 7; Spectrum Laboratories, Rancho Dominguez, CA). The purity and structure of the final products were verified respectively by an analytical C18 RP HPLC (Beckman Coulter GOLD System, Brea, CA) and MALDI mass spectroscopy (Voyager Elite; PerSeptive Biosystems, Framingham, MA). Before treatment, NTCIs (cSN50.1 and FITC-cSN50.1 peptides) were solubilized in sterile water (half the final volume) and diluted with sterile saline to a final concentration of 3.3 mg/ml (cSN50.1) and 4.1 mg/ml (FITC-cSN50.1).

### 2.2 Preparation of cecal microbiome (CM) stock

8 – 10-week-old C57BL/6 mice (Jackson Laboratories, Bar Harbor, ME) were euthanized by over inhalation of isoflurane followed by cervical dislocation. The cecum was removed, and the gut microbiome content was extruded into a separate pre-weighed vial according to the published method (18, 32) modified as follows. The collected cecal contents were weighed, and mixed with 5% dextrose (D5W) to final concentration of 80 mg/ml. The cecal content was strained through a 70 μm mesh strainer (VWR, Radnor, PA). The filtered cecal microbiome (~250 µl/vial) was frozen at -80°C as CM stock. To determine the stock’s bacterial count (CFU/ml), 20 µl of fresh CM stock from each tube was diluted 1:100 with sterile PBS, and 100 µl of diluted stock mixture was cultured on Tryptic Soy Agar (TSA) + 5% sheep blood plates (Thermo Fisher Scientific, Hampton, NH) and incubated overnight at 37 ˚C for the count of non-viridans bacterial colonies. For experimental consistency, the tubes with bacterial count ranging from 4.0×10^4^ to 5.0×10^4^ CFU/ml were kept stocked. Before each intraperitoneal injection, several vials were combined to suffice injection of 4.5×10^5^ CFUs/kg. In parallel, a sample of 20 µl from pooled CM stock was tested for quality control, as described above.

### 2.3 Animal studies

Animal experiments were carried out in compliance with the ARRIVE guidelines and in strict conformity with the Guide for the Care and Use of Laboratory Animals of the National Institutes of Health. The submitted protocols were approved by the Vanderbilt University Institutional Animal Care and Use Committee. In all animal assays, an adapted model of nonsurgical polymicrobial peritonitis was used (18, 32). During the course of experiments, mice were closely monitored and euthanized by isoflurane over inhalation followed by cervical dislocation upon expression of the signs of moribund state. Survivors were euthanized at the experimental end point. The experimental groups were selected using a double blinded randomization method (21). Each experiment was performed at least twice to assure statistical significance and experimental reproducibility. Blood was collected from retroorbital plexus under isoflurane anesthesia shortly before euthanasia.

#### 2.3.1 Infection and treatment

The experimental groups (Sham Control + Saline, n = 5; CM-Challenged + Saline, n = 5; and CM-Challenged + NTCI, n = 5) were comprised of randomly assigned 8 – 10-week-old female C57Bl/6J mice (The Jackson Laboratory, Bar Harbor, ME). The mice were infected by single intraperitoneal (i.p.) injection of a CM stock corresponding to 4.5×10^5^ CFU/kg (between 9 - 11.5 µl/g of CM stock). For NGS, qRT PCR, and cytokines analyses, mice were treated with the i.p. injection of NTCI (cSN50.1 peptide, 33 μg/g/injection in 200 μl 0.45% NaCl) or 0.45% NaCl (200 μl) as vehicle control, at 30 min before and 30 min, 1.5h, 2.5h, 3.5h, and 5h after CM challenge. To determine the direct effect of NTCI treatment on gene expression in lungs and kidneys uninfected, mice were treated with NTCI or vehicle control, following the same injection schedule. For determination of bacterial dissemination in the blood, lungs, spleen, and kidneys, the NTCI and saline treatments were adjusted as follows: 30 min before and 30 min, 1.5h, 2.5h, 3.5h, 6h, and 9h after CM challenge. Mice were euthanized at 6h for genomic analysis and at 12h post-CM challenge for bacterial dissemination study.

#### 2.3.2 Determination of LD_50_ for cecal microbiome (CM)

Randomly grouped (n = 6) 8-10-week-old female C57Bl/6J mice (The Jackson Laboratory, Bar Harbor, ME) were infected by single i.p. injection of a CM stock corresponding to 7.5×10^5^, 9.0×10^5^, 1.05×10^6^ or 1.2×10^6^ CFU/kg. Mice were closely monitored throughout the experiment and euthanized upon expression of the signs of moribund state. Survivors were euthanized at 168 h post CM challenge.

### 2.4 Gene expression profiling by qRT PCR

Mice were euthanized at 6 hrs. post CM challenge and isolated lungs were disrupted in lysis buffer on ice using a Dounce hand homogenizer. Total RNA was isolated using NucleoSpin RNA Plus kit (Macherey-Nagel, Germany) according to the manufacturer’s instructions. RNA concentration and purity were determined using a NanoDrop One spectrophotometer (Thermo Fisher Scientific, Hampton, NH). The 1 µg of total RNA was reverse-transcribed using iScript cDNA synthesis kit (Bio-Rad, Hercules, CA). A real-time quantitative reverse transcription PCR was carried out in a 96-well plate on a QuantStudio 3 instrument (Thermo Fisher Scientific, Hampton, NH) using Taqman Fast Advanced Master Mix (Thermo Fisher Scientific, Hampton, NH), according to the manufacturer’s protocol. FAM-labeled probes for mouse genes were obtained from Thermo Fisher Scientific. The raw Ct values were converted into relative expression levels using Livak methods (2^−ΔΔCt^) with 18S gene as reference and Mock Control (unchallenged and not treated mice) as calibrator (control). Converted Ct values were used for statistical analysis.

### 2.5 Next-generation sequencing (NGS)

Mice were euthanized at 6 hrs. post CM-challenge and the lung and kidney tissues were processed for total RNA extraction as described above. Three samples from each experimental conditions (Sham + Saline, CM + Saline, CM + cSN50.1, and cSN50.1 w/o CM) from lungs and kidneys were submitted to the Vanderbilt Technologies for Advanced Genomics (VANTAGE) Core for RNA sequencing analysis. RNA integrity numbers (RIN) were measured using TapeStation system (Agilent Technologies, Santa Clara, CA) and total RNA was processed into Stranded mRNA (NEB) Library. RNA sequencing was performed using Illumina NovaSeq6000 (San Diego, CA). The quality control of RNA preparation was analyzed by DRAGEN RNA Pipeline (v3.7.5). The differential expression of the genes was analyzed using Illumina DRAGEN Secondary Analysis software. The expressions of approximately 17 to 18 thousand genes were analyzed in each organ and condition. Number of genes considered as significantly expressed were determined by a false discovery rate (FDR) set to *p*_adj_<0.05. Genes with increased/decreased expression, as compared to the experimental control group, were selected from the pool of genes significantly expressed displaying values of log_2_(fold change) grater/less than center of distribution ± standard deviation determined by Gaussian distribution plot (**Suppl. Figure SF3**). Genes with log_2_(FC) values located within the range established by standard deviation were considered as genes with unchanged expression.

The analyzed conditions were: CM + Saline vs. Sham + Saline to evaluate the impact of sepsis on gene expression, and CM + NTCI (cSN50.1) vs. CM + Saline to evaluate the impact of selective nuclear blockade on sepsis-induced gene expression. The condition of NTCI vs. Saline were used to determine an effect of NTCI treatment on gene expression in healthy animals without sepsis.

### 2.6 Determination of bacterial dissemination in blood, lungs, spleen and kidneys

Mice were euthanized 12 hours post CM challenge (see Animal Studies for treatment details) and blood, spleen, lungs, and kidneys were collected for the analysis of bacterial count. Blood (50 µl) was diluted 1:20 with sterile PBS and 200 µl of diluted blood sample was applied to TSA + 5% sheep blood agar culture dish and incubated overnight at 37 ˚C. Isolated organs were externally sterilized by brief immersion in 70% ethanol, washed with cold sterile PBS, and placed in pre-weighed vials containing 0.5 ml cold sterile PBS. Tissue samples were weighed, and net weights were recorded. Organs were homogenized with a disposable sterile plastic homogenizer and suspension passed through the 70 µm cell strainers. 50 µl of suspension was serially diluted with sterile PBS resulting in the final concentration of 1:100 (lungs), 1:10,000 (spleen), and 1:500 (kidneys). The diluted samples (200 µl) were applied to TSA + 5% sheep blood agar plate for overnight incubation at 37˚C. The colonies (non-viridans) were counted down and then converted into CFU/ml (blood) or CFU/g of a wet organ mass (lungs, spleen, and kidneys).

### 2.7 Cytokine assay

The proteins levels of cytokines IL-6, IL-10, TNF-α, and IFN-γ were measured in blood plasma collected postmortem from the hearts of mice euthanized at 6 hrs. post CM/Sham challenge (groups Sham + Saline, CM + Saline, and CM + cSN50.1) or 1 hr. after last NTCI treatment (cSN50.1 only). A cytometric bead array (BD Biosciences) assay was performed and analyzed in the Vanderbilt University Medical Center Flow Cytometry Shared Resource as previously described (18).

### 2.8 Kirby-Bauer antimicrobial susceptibility test (AST)

To determine the NTCI’s potential antibacterial activity, the indicated amounts of peptide (in 10 µl aliquots) were loaded up to the blank sterile discs (Thermo Scientific™ Oxoid™, Thermo Fisher Scientific, Hampton, NH) using 1 mM or 5 mM stock solution. To allow solvent evaporation, disks, under sterile conditions, were flipped over several times between loads until completely dry. Kirby-Bauer AST was performed using meropenem-loaded disks (10 µg and 30 µg) as positive control. CM was diluted 1:10 and 1:20 with PBS and 200 µl inoculum suspension was spread evenly on TSA + 5% sheep blood agar plates. NTCI- or meropenem-loaded discs were placed on inoculated agar plates and pressed gently down to ensure contact with the agar surface. The plates were placed in an incubator set to 37 ˚C. After 24 hours of incubation plates were examined by measuring the diameters of the complete inhibition zones.

To determine the NTCI’s diffusion zone from the disks into agar medium, a 300 nmol of FITC-labeled NTCI was loaded on the blank disc. Disc was applied to the surface of the regular agar plate for overnight incubation at 37 ˚C. FITC-labeled NTCI diffusion into the agar gel was determined by fluorescent imaging (Gel Doc EZ imaging system; Bio-Rad, Hercules, CA).

### 2.9 Statistical analysis

Normal distribution of data sets was verified using normal probability plot (q-q) and Kolmogorov-Smirnov Normality Test. A statistical analysis was performed using Prism 6 software (GraphPad, Boston, MA). Gene expression profile by qRT PCR of lungs total RNA samples and plasma levels of cytokines were analyzed by ordinary one-way ANOVA with an uncorrected Fisher’s LSD test for a multiple comparison. Bacterial dissemination in blood, lungs, spleen and kidneys was analyzed by nonparametric t test with Mann–Whitney rank comparison. The data is presented as a mean ± SEM. *p* values of < 0.05 were considered significant. The standard deviation and the center of the log_2_(FC) values distribution in NGS analyzed data was determined by the Gaussian plot (**Suppl. Figure SF3**). Log_2_(FC) values were rounded down to the nearest decimal point (0.1) and equal values were added up. These numbers were then plotted against log_2_(FC) and analyzed by a Gaussian/Lorentzian function using Prism 6 software (GraphPad, Boston, MA).

## 3 Results

### 3.1 Sepsis induces the genes encoding mediators of inflammation in the lungs while the selective nuclear blockade with the NTCI suppresses this induction as detected by the two independent methods [quantitative PCR and the next generation sequencing (NGS)]

**Figure 1:**
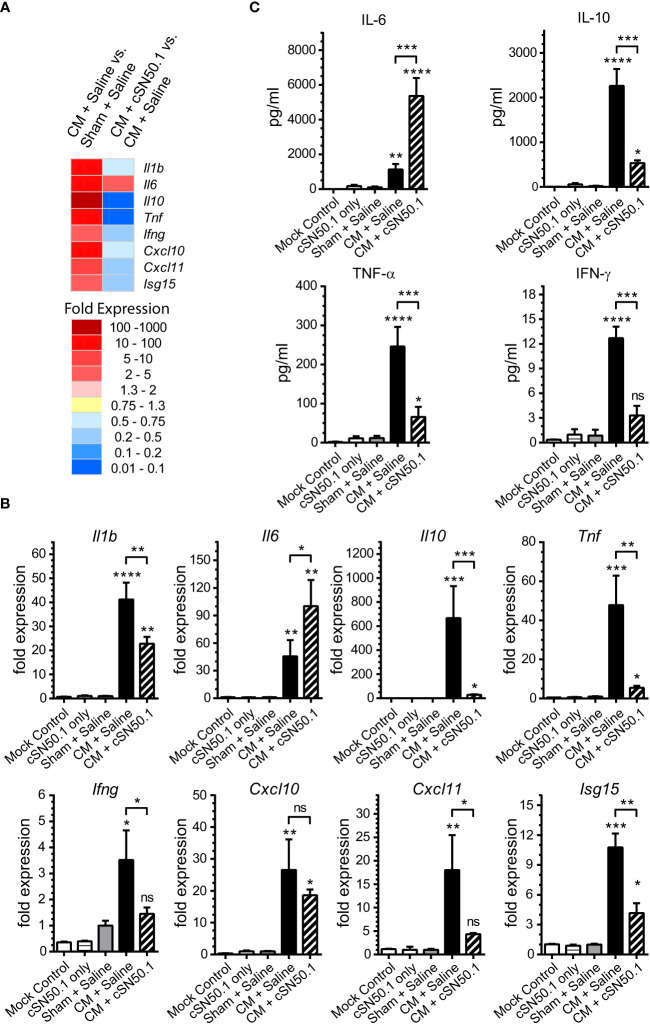
The 8 Genes Encoding Mediators of Innate Immunity and Inflammation Were Selected for the Comparison of the Two Methods of Genomic Analysis of the Lungs in Sepsis. The selected genes display strong response to sepsis as detected by the NGS **(A)** and verified by real-time quantitative reverse transcription PCR (Panel B). The relative levels of expression in qRT PCR **(B)** were established using Livak’s methods (2^−ΔΔCt^) with the 18S gene as a reference and the Mock Control (unchallenged and not treated mice) group as the calibrator (control). Data is presented as a mean + S.E.M. (n=3 for Mock Control, n=5 for all other conditions). **(C)** The level of cytokines IL-6, IL-10, TNF-α, and IFN-γ was determined in blood plasma collected postmortem from hearts of mice euthanized 6h after CM (or Sham) challenge (groups Sham + Saline, CM + Saline, and CM + cSN50.1) or 1 hr. after last NTCI treatment (cSN50.1 only). Data is presented as a mean ± SEM (n = 5). The selective nuclear blockade with the NTCI suppressed expression of 7 analyzed genes while the gene encoding IL-6 remained increased after the NTCI treatment, as determined by the two methods of genomic analysis and confirmed by proteomic analysis in blood plasma (see Materials and Methods for details). A statistical analysis in Panels B and C was performed using an ordinary one-way ANOVA with an uncorrected Fisher’s LSD test for a multiple comparison, *p < 0.05, **p < 0.005, *** *p* < 0.0005, ****p < 0.0001. The significance levels displayed over the error bars of CM + Saline and CM + cSN50.1 columns represent the statistical difference of each compared to Sham + Saline samples.

The lungs and kidneys are the major organs that fail during sepsis (5). Therefore, we focused first on the lungs, the site of ARDS, a severe complication of infections (23, 24). In the polymicrobial sepsis, we compared the expression of the selected genes encoding mediators of inflammation analyzed by the quantitative PCR, to that determined by the NGS (**Figures 1A, B**). The 8 representative genes encoding cytokines and chemokines (IL-1β, IL-6, IL-10, TNF-α, IFN-γ, CXCL10, CXCL11), and ISG15 (interferon-stimulated gene 15, which encodes the ubiquitin-like modifier of intracellular signaling proteins), were consistently induced in the lungs during the experimental sepsis. The NTCI (cSN50.1 peptide) suppressed their expression, as analyzed by both genomic methods (**Figures 1A, B**). One notable exception was the gene encoding the pleiotropic cytokine, IL-6, which remained elevated in the NTCI-treated mice. The consistency of our results based on the two methods of genomic analysis was further supported by the proteomic analysis of the corresponding changes in the blood plasma proteins encoded by the selected genes (**Figure 1C**). As expected, the protein levels of IL-10, TNF-α, and IFN-γ were increased in the CM-challenged mice. The treatment with NTCI (cSN50.1 peptide) significantly reduced their concentration reflecting their cognate gene suppression in the lungs (**Figures 1A, B**). Contrariwise, the IL-6 protein level remained elevated in the plasma of the NTCI-treated animals (**Figure 1C**). Together, the concordance of the two methods of genomic analysis was also consistent with the proteomic analysis of plasma proteins. Reassured by these results, we embarked on the comparative study of the genomic response to sepsis in the two major target organs, lungs and kidneys, using the NGS.

### 3.2 The sepsis-induced genes encoding mediators of inflammation in the lungs and kidneys are controlled by the selective nuclear blockade with the NTCI

**Figure 2:**
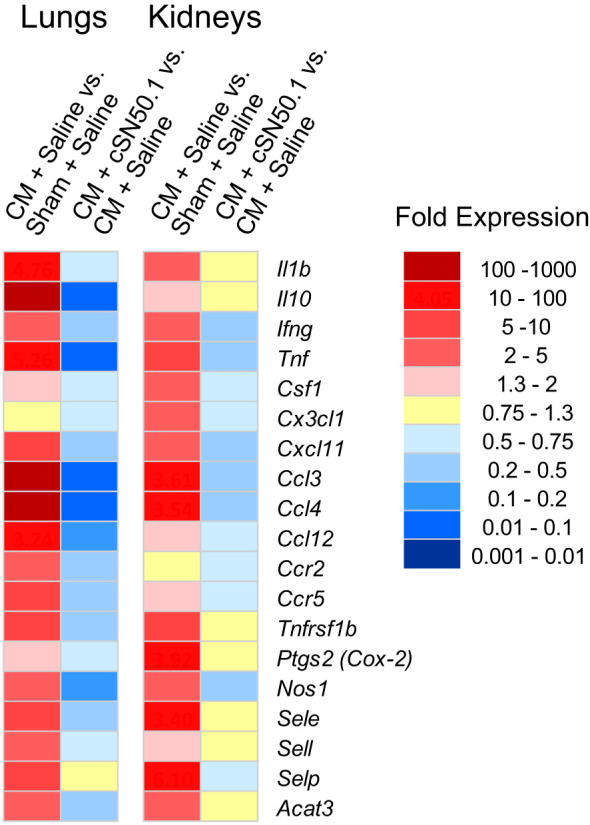
Similar Expression of Genes Encoding Cytokines, Chemokines and Signaling Intermediates in the Lungs and Kidneys, and their Less Robust Decrease in the Kidneys of the NTCI-Treated Animals. The NGS analysis of total RNA samples extracted from lungs and kidneys of CM-challenged mice treated with the NTCI for 6 hours (n = 3, see Materials and Methods for details). Please note that elevated genes in the lungs were particularly susceptible to the nuclear blockade with the NTCI. ns means not significant.

The first group of the 19 genes encoding the known mediators of inflammation in the lungs and kidneys included the following: (i) genes expressing cytokines: *Il1b*, *Tnf*, *Ifng*, *Il10*; (ii) chemokines: *Ccl3*, *Ccl4*, *Ccl12*, *Ccr2*,*Ccr5*; (iii) hemopoietic growth factor (*Csf1*), also known as granulocyte/monocyte colony-stimulating factor; (iv) cell adhesion molecules: selectins (endothelial, platelet, and leucocyte); and (v) genes encoding signal transducers: cyclooxygenase-2, nitric oxide synthase-1, and cholesterol acyltransferase 3 (*Acat3*), the catalyst in the cholesterol esters synthesis (33). As shown in **Figure 2**, among these 19 representative genes in the lungs and kidneys, 12 displayed very similar pattern of expression in both organs whereas *Csf1*, *Cx3cl1*, and *Ptgs2* (Prostaglandin-endoperoxide synthase 2, aka Cox2) showed higher expression in the kidneys. The treatment with the NTCI dramatically counteracted the activation of these genes in both organs. Remarkably, the six sepsis-elevated genes in the lungs (*Il10, Tnf, Ccl3, Ccl4, Ccl12*, and *Nos1*) were more susceptible to the nuclear blockade with the NTCI than their homologs in the kidneys (**Figure 2**). The cellular and molecular architecture of the two organs may contribute to their different genomic response to sepsis (see Discussion).

### 3.3 Sepsis causes massive genomic response in the lungs and kidneys that is countered by the NTCI

**Figure 3:**
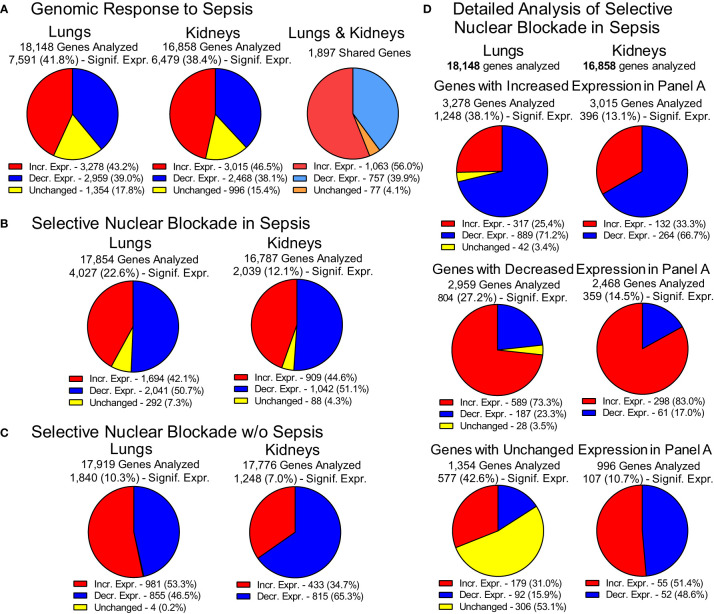
Genomic Response to Polymicrobial Sepsis in the Lungs and Kidneys. **(A)** The response of lungs and kidneys’ genome to sepsis. Red – genes with increased expression, blue – genes with decreased expression, yellow – genes with unchanged expression. Please note that the pie charts are constructed based on genes significantly expressed (*p*_adj_ < 0.05, see Materials and Methods for details). The 1,897 genes displayed similar pattern of expression in both organs. These genes are termed “common” or “shared” or organ-non-specific genes in the manuscript. **(B)** The overall view of the gene expression in the lungs and kidneys of mice following the Selective Nuclear Blockade with cell-penetrating NTCI (cSN50.1 peptide). **(C)** Impact of NTCI treatment on gene expression in control animals without sepsis. **(D)** Detailed analysis of the Selective Nuclear Blockade of the increased, decreased or unchanged pools of genes in sepsis as presented in Panel A (see text for details).

The impact of experimental sepsis on the transcriptome in the lungs and kidneys was astounding (**Figure 3**). In the lungs, among the 18,148 analyzed genes comprising the lung transcriptome, 7,591 (41.8%) genes were significantly expressed during sepsis. Within this set, 3,278 genes (43.2%) displayed increased expression and 2,959 genes (39%) showed decreased expression whereas 1,354 genes (17.8%) remained unchanged. In the kidneys, among the 16,858 analyzed genes comprising the kidneys transcriptome, 6,479 (38.4%) showed significant expression. Within this set, the expression of 3,015 genes (46.5%) was increased and 2,468 genes (38.1%) was decreased in response to sepsis (**Figure 3A**). The expression of 996 (15.4%) genes did not show significant difference from uninfected control. The increased expression of 1,063 genes was observed in both organs whereas the expression of 757 genes was decreased in lungs and kidneys. In total, 1,897 genes shared common pattern of expression in the two organs.

This extraordinary genomic response to sepsis in the two major organs was profoundly changed by the selective nuclear blockade with the NTCI during the 6-hour treatment (**Figure 3B**). Among 17,854 genes analyzed in the lungs and 16,787 genes analyzed in the kidneys, 4,027 genes were significantly expressed in the lungs and 2,039 genes in the kidneys. The impact of the NTCI treatment on the gene expression in the lungs and kidneys was bidirectional. In the lungs, 1,694 (42.1%) genes were increased and 2,041 genes (50.7%) were decreased whereas 292 genes (7.3%) were unchanged during selective nuclear blockade with NTCI. In the kidneys, the expression of 909 (44.6%) genes was increased and 1,042 (51.1%) genes were decreased while the expression of 88 (4.3%) genes remained unchanged during selective nuclear blockade with NTCI (**Figure 3B**).

We also considered the potential impact of NTCI on the gene expression in control mice not challenged with sepsis-producing cecal microbiome (CM) (**Figure 3C**). The NGS analysis of selective nuclear blockade by NTCI in control animals without sepsis showed that among 10.3% genes significantly expressed in the lungs (1,840 of 17,919 analyzed), 981 (53.3%) showed an upward and 855 (46.5%) displayed downward expression. In the kidneys, where only 7% of analyzed genes were significantly expressed (1,248 of 17,776 analyzed), the majority, 815 genes (65.2%), showed downward regulation, whereas expression of 433 genes (34.7%) was increased (**Figure 3C**). Genomic response in animals without sepsis treated with the multiple intraperitoneal injections of the NTCI or saline (control) could be partially attributed to the stress associated with repeated interventions.

The detailed analysis of gene expression in infected animals treated with NTCI (**Figure 3D**) showed that selective nuclear blockade with cSN50.1 decreased expression of 889 genes in the pool of genes increased by sepsis in the lungs (Panel A). Likewise, the NTCI treatment reduced expression of 66.7% genes in the pool of genes increased in the kidneys. In contrast, the expression of 589 lung genes and 298 kidney genes that were decreased in sepsis were strikingly increased by the NTCI (73.3% and 83.0%, respectively). These results in the animals with polymicrobial sepsis document the normalizing effect of the NTCI on genomic response to the microbial inducers and the host-produced mediators of inflammation.

Thus, we found that experimental sepsis elicits the enormous mobilization of the genome in the lungs and kidneys. The virulence factors of sepsis-causing microbial agents together with the host-produced mediators of inflammation, massively activated inflammatory and metabolic regulome in the cell nuclei of the lungs and kidneys. Our findings of this unprecedented “genomic storm” in the lungs and kidneys advance the understanding of sepsis through the identification of the genes displaying common pattern of expression, shared by both organs, and the genes displaying organ-specific expression in the lungs and kidneys involved in response to sepsis (see below). Moreover, we expanded this analysis to the genes controlled by the selective nuclear blockade with the NTCI in the lungs and kidneys of infected animals (see below).

### 3.4 The top 100 shared genes responding to sepsis in the lungs and kidneys that are regulated by the NTCI

The roster of the top 100 genes changed by sepsis in the lungs and kidneys as compared to the genes analyzed in the unchallenged “sham” controls treated with saline is presented in the **Supplemental Table 1, ST1A**. The 11 most sepsis-upregulated genes in the lungs differed in their maximal response from that of the kidneys. Nevertheless, there was a similar pattern of their inhibition by the NTCI with the notable exception of the gene encoding Colony Stimulating Factor 3 (granulocyte, *Csf3*), which was not suppressed by the NTCI in both organs. The gene encoding Interleukin 10 (*IL10*) was robustly responding to sepsis in the lungs being reduced almost 20-fold by NTCI albeit the almost 70-fold lower expression in the kidneys was unchanged by NTCI. Similarly, the highly elevated expression of the gene encoding chemokine (C-X-C motif) ligand 2 (*Cxcl2*) was reduced by the NTCI in the lungs but increased in the kidneys.

Strikingly, the *Saa3* gene encoding the acute phase protein response biomarker (Serum Amyloid A-3), was increased by sepsis in the lungs (412-fold) as compared to the kidneys (23-fold) (**Suppl. Table ST1A**). Whereas the role of cytokines and chemokines in sepsis is generally known (see above), the impact of elevated serum amyloid proteins A1-A3, as acute phase proteins akin to the C-Reactive Protein in humans, came to fore recently. The serum amyloid proteins A1-A3 contribute to muscle atrophy, known as the Intensive Care Unit-Acquired Weakness (ICUAW) delaying patients’ recovery. Serum Amyloid A1 is recognized by the Toll-like Receptors 2 and 4 triggering nuclear signaling mediated by NF-κB and leading to the myotube injury (34). Importantly, the treatment with the NTCI lowered *Saa3* gene expression four-fold in the lungs and two-fold in the kidneys.

The gene encoding aconitate decarboxylase (*Acod1*), also known as the immune responsive gene 1 (IRG1) was strikingly induced 452-fold by sepsis in the lungs whereas the NTCI attenuated its expression to the 103-fold level over the untreated control. In comparison, the same gene in the kidneys was induced by sepsis less robustly (73-fold) while the NTCI reduced its expression to 53-fold level. The product of IRG1, itaconate, suppresses succinate dehydrogenase (35) while accumulation of succinate controls adipose tissue thermogenesis (36). Moreover, itaconate causes the electrophilic stress mobilizing the transcription factors NRF2 and ATF3 (37, 38). Nuclear translocation of NRF2 is mediated by Importin α5 (39) targeted by the NTCI (20). Thus, NTCI not only suppresses the generation of itaconate but also its electrophilic stress-induced nuclear signaling mediated by NRF2 (19).

Among the analyzed “common genes” shared between the lungs and kidneys is the gene encoding Fos-like antigen (*Fosl1*), a component of the AP1 transcription factor complex that belongs to the Stress-Responsive Transcription Factors (19). It was upregulated by sepsis 48-fold and 11-fold in the lungs and kidneys, respectively. The NTCI treatment downregulated its expression to the 31-fold level in the lungs. Contrariwise, expression of *Fosl1* gene in the kidneys was increased two-fold by NTCI. Importantly, in addition to changing the expression of *Fosl1*, the NTCI inhibited nuclear translocation of FOSL1 bound to cJun (AP1 protein complex) during sepsis (18). FOSL1 was also identified in transcriptomic analysis of differentially expressed genes in SARS-Co-2 infected lung epithelial cells as compared to the pancreatic islet cells (40).

Genes encoding members of the acute phase protein response, C-reactive protein (*Crp*), and orosomucoid 1 and 2 (*Orm1, Orm2)* belong to the group of genes upregulated by sepsis in both organs (41). The gene encoding *Orm1* was tightly regulated by NTCI treatment, especially in the kidneys, where its expression was reduced almost to the physiologic levels. Similarly, three genes encoding fibrinogen alpha (*Fga*), beta (*Fgb*), and gamma chains (*Fgg*), and Coagulation Factor X (*F10*) were increased by sepsis in the lungs and kidneys. Coagulation Factor X and fibrinogen, the main blood clotting protein, are also elevated during the aforementioned acute phase protein response contributing to the blood hypercoagulability known as thrombophilia (42). In combination with the microvascular endothelial injury, it evolves into disseminated intravascular coagulation (DIC), one of the major hematologic complications of sepsis (5). The NTCI treatment reduced the expression of the genes encoding fibrinogen chains and Factor X in the lungs and kidneys thereby potentially reducing the risk of DIC in sepsis, and by extension, in thrombophilia. Consistent with the current findings, the NTCI reduced the signs of massive microvascular liver injury and the laboratory signs of DIC (43).

We also spotted the genes that are not controlled by the NTCI treatment among the top responders to sepsis in the lungs and kidneys. Besides *Csf3*, this group encompasses metallothionein 2 (*Mt2*), interleukin 1 receptor type II (*Il1r2*), neutrophilic granule protein (*Ngp*), matrix metallopeptidase 8 (*Mmp8*), interleukin 6 (*Il6*), chemokine (C-X-C motif) ligand 1, 2 and 3 (*Cxcl1, Cxcl2, Cxcl3*), and CD177 antigen (*Cd177*).

The most downregulated genes by sepsis in the lungs and kidneys feature anoctamin 5 encoding gene *Ano5* (see **Suppl. Table ST1B**). Its product comprises a 913-amino acid protein of the anoctamin family that is involved in phospholipid scrambling required for the sarcolemma repairing process in the skeletal muscles. Mutations in *Ano5* give rise to an autosomal dominant skeletal muscle myopathies and dystrophies (44). It is likely that the suppression of anoctamin 5 gene in sepsis may contribute to the aforementioned Intensive Care Unit-Acquired Weakness (ICUAW) linked to the elevated Amyloid A-1-3 in sepsis (34). NTCI treatment raised 5-fold the expression of Anoctamin 5 gene while reducing significantly Amyloid 1-3 genes in sepsis (see **Suppl. Tables ST1A, ST2A**).

The NTCI also increased the expression of the gene encoding oxytocin receptor (*Oxtr*), a 7-transmembrane G protein-coupled receptor that activates a set of signaling cascades, such as the MAPK, PKC, PLC, or CaMK pathways, which converge on transcription factors CREB or MEF-2 (45). OXTR regulates neurite outgrowth, cellular viability, and increased survival. NTCI partially restored the *Oxtr* gene expression in the lungs and kidneys (**Suppl. Table ST1B**). Likewise, NTCI increased another sepsis-downregulated gene encoding apelin receptor (*Aplnr*) in the lungs but decreased its expression in the kidneys (**Suppl. Table ST1B**). Significantly, in cellular signaling, apelin is an endogenous peptide ligand for the APJ receptor involved in angiogenesis, cellular homeostasis, cardiovascular maintenance, and neuroprotection. In response to apelin, the APJ receptor drives the PI3K/Akt, MAPK, and PKA signaling pathways, leading to cell proliferation and protection from excitotoxicity (46). Similarly, the gene encoding β-Klotho was downregulated by sepsis in the lungs and kidneys and increased by the NTCI. This essential component of fibroblast growth factor (FGF) receptor complexes is required for high-affinity binding of endocrine FGF19 and FGF21 to evoke the signaling cascade actively involved in homeostatic maintenance of glucose metabolism and energy expenditure that are dysregulated in sepsis (47).

### 3.5 The top 100 most responsive lung genes in sepsis and their regulation by the selective nuclear blockade with the NTCI

Next, we focused on the top 100 most upregulated and 100 most downregulated genes by sepsis in the lungs and determined the impact of the selective nuclear blockade with NTCI on their expression.

Among the most sepsis-upregulated genes in the lungs, we noted the top 14 genes displaying remarkable rise of 119-fold to 3,985-fold in their upward expression (the shades of red color of increased intensity up to the brown color in the heat map, respectively in response to sepsis) shown in **Suppl. Table ST2A**. Three of the most upregulated genes encode *Saa2, Saa1*, and *Prok2*. Whereas serum amyloid genes (*Saa1, Saa2, and Saa3)* were addressed earlier, *Prok2* (upregulated 1,783-fold), that encodes prokineticin 2 is also noteworthy. Its deficiency is linked to the low levels of gonadotropins and testosterone with or without anosmia (48). The NTCI treatment reduced its expression to 450-fold level while the gene encoding Prok2 receptor (*Prokr2)* was reduced to 4.5-fold level by the NTCI. The role of prokineticin 2 and its receptor in the development of ARDS and other lesions of acute lung injury in sepsis is unknown although anosmia is a prominent feature of SARS Coronavirus-2 infections (49).

Two other genes strongly responding to sepsis are of particular interest: *Tarm1* and *Mrgprb2.* The former encodes T cell-interacting activating receptor on myeloid cells. This novel leukocyte receptor expressed in neutrophils and macrophages plays an important role in proinflammatory response to acute bacterial infections. Simultaneous stimulation of TARM-1 and Toll-like receptor 4 enhanced the production of TNFα and IL-6 by granulocytes and macrophages (50). In turn, *Mrgpra2a* is the homolog to the human gene encoding MRGPRX2. This important protein mediates mast-cell activation in response to a broad array of stimuli ranging from wasp venom to several pharmaceutical compounds associated with the IgE-independent pseudo-allergic reactions in patients (51). The NTCI treatment reduced its expression in the lungs (**Suppl. Table ST2A**).

Among the most downregulated lung genes in sepsis, is defensin beta 42 (*Defb42*) displaying predominantly anti-bacterial activity against *Escherichia coli* and *Staphylococcus aureus* (52). The expression of this gene in the lungs of NTCI-treated mice was not significant.

### 3.6 The top 100 most responsive kidney genes in sepsis and their regulation by the selective nuclear blockade with the NTCI

Among the top 100 most upregulated genes by sepsis in the kidneys (see **Suppl. Tables ST3A, ST3B**), is eosinophil peroxidase (Epx) which displayed 118-fold increase. It was almost doubled by the NTCI to 231-fold level. As no clinical findings have been associated with human *Epx* deficiency (53), its overexpression in the kidneys during sepsis awaits further studies. In turn, G protein-coupled bile acid receptor-1 (*Gpbar1* aka Takeda G protein-coupled receptor-5 [TGR5]) regulates bile acid metabolism and glucose and insulin sensitivity. TGR5 stimulates glucagon-like peptide-1 (GLP-1) secretion to improve insulin sensitivity and hepatic metabolism (54). Significantly, the treatment with the NTCI increased 4-fold the expression of the TGR5 underscoring the potential for metabolic improvement in sepsis.

Among the genes in the kidneys displaying extreme downregulation during sepsis, the two genes encoding cytochrome P450, family 11 subfamily b and a polypeptide 1 were restored by the NTCI to higher albeit still abnormal levels. Along with the genes encoding cytochrome P450 family 17 subfamily A member 1, they participate in steroidogenesis being induced by endothelin-1 (55). The profound suppression of these genes indicates potential failure of physiologic steroidogenesis in sepsis.

### 3.7 Enhancement of bacterial clearance in polymicrobial sepsis by the selective nuclear blockade

As indicated above, sepsis had a significant impact on the genes encoding multiple mediators of innate immunity and inflammation in the lungs and kidneys, including Solute Carrier Family 11 (Proton-Coupled Divalent Metal Ion Transporters, Member 1 (*Slc11a1*), aka natural resistance associated macrophage protein 1 (NRAMP). This gene is linked to the control of intracellular pathogens, and along with the gene encoding cathelicidin antimicrobial peptide (*Camp*) and defensins, contributes to the innate immunity-mediated bacterial clearance in the lungs and kidneys as well as blood and spleen (see below). This process depends on the phagocytic system (56).

**Figure 4:**
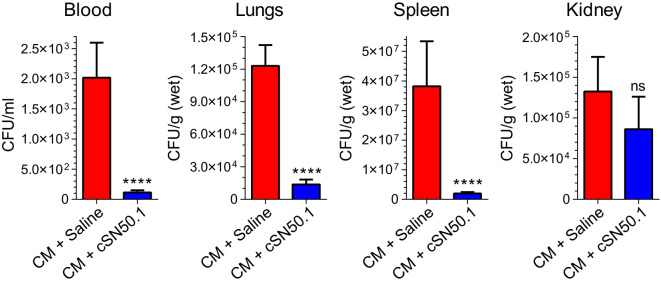
Selective Nuclear Blockade with the NTCI Significantly Reduced Bacterial Dissemination in Blood, Lungs, Spleen, and Kidneys. Mice with induced polymicrobial peritonitis were treated for 12 hours with the NTCI without antimicrobial therapy (see Materials and Methods for details). Blood, lungs, spleen, and kidneys were collected and processed for bacterial count (n=5). Bacterial dissemination in blood and three major organs was analyzed by nonparametric t test with Mann–Whitney rank comparison. The data is presented as a mean ± SEM. ****p < 0.0001, ns, not significant.

To study bacterial clearance in sepsis, we adopted its clinically relevant experimental model evolving from polymicrobial peritonitis. This non-surgical model provides consistent results in terms of the standardized microbial challenge as compared to the alternative surgical model, the cecal ligation and puncture. The latter is subject to the uncontrolled spillage of cecal microbiome into the peritoneal cavity. The cecal microbiome (abbreviated CM) was obtained from the euthanized healthy donor mice (18). The gut microbiome of healthy C57Bl/6 mice comprises: Dorea, Actinobacteria, Bifidobacteriaceae, Turucibacteriaceae, Firmicutes, Lactobacilliaceae, and other Bacilli (57). The mice were uniformly infected with a titrated amount of gut microbiota through a single intraperitoneal injection (4.5×10^5^ Colony Forming Units (CFU)/kg, representing half of the LD_50_ determined for Cecal Microbiome (CM) (**Suppl. Figure SF1**). We hasten to add that this microbial challenge comprises a multitude of intestinal Gram-negative bacteria expressing diverse LPS structures and additional virulence factors, Gram-positive bacteria, and other microbes residing in the gut microbiome (18, 32). Importantly, the resulting polymicrobial peritonitis is accompanied by dissemination of the infection to the blood (bacteremia), lungs, spleen, kidneys, and other organs (**Figure 4**), thereby producing a multi-system microbial inflammation that mediates sepsis.

Strikingly, the selective nuclear blockade with the parentally administered NTCI for 12 hours (without antimicrobial therapy) reduced the detectable bacterial count in the following ways: 18-fold in the blood, 9-fold in the spleen, 11-fold in the lungs (**Figure 4**). The downward change of the bacterial count in the kidneys was not significant. We link these NTCI-induced gains in the bacterial clearance in the lungs, as compared to the kidneys, to the different genomic response to polymicrobial sepsis in these two target organs. We excluded the direct action of the NTCI on bacterial population comprising CM by using the Kirby-Bauer method of bacterial sensitivity testing with antibiotic meropenem as the positive control (**Suppl. Figure SF4**). Previously, we showed that the addition of NTCI to meropenem, a broad-spectrum β-lactam antibiotic, increased survival to 55% in a similar model of sepsis whereas meropenem alone afforded 30% survival (18).

## 4 Discussion

We report that polymicrobial sepsis in the preclinical model causes a massive genomic response in the two main target organs, the lungs and kidneys. This unprecedented, major organ-based genomic response is attenuated by the selective nuclear blockade with the NTCI. Astoundingly, 3,735 sepsis-induced genes in the lungs and 1,951 sepsis-induced genes in the kidneys were reprogrammed by 6-hour treatment. Moreover, the NTCI-treated animals displayed the enhancement of microbial clearance that reduced the sepsis-causing dissemination of titrated gut microbiome following its intraperitoneal injection. The bacterial clearance was increased 18-fold in the blood, 9-fold in the spleen, and 11-fold in the lungs during the 12-hour treatment with the NTCI. Importantly, the 9-fold increase in microbial clearance in the spleen is clinically relevant. Patients with hyposplenism due to celiac disease or following bone marrow transplantation are at a significant risk of fulminant sepsis (58). Thus, the data sets presented herein and their impact on bacterial clearance in experimental sepsis serve as a translational platform for future clinical studies of patients with sepsis. Ultimately, the massive genomic response to sepsis, which is treated by the selective nuclear blockade, provides a foundation for much needed new therapeutic approaches. Our data also provide a basis for evaluation of the microbial clearance in other model systems to address the host’s genomic response to the sepsis-causing viral, fungal, and protozoal pathogens. The prevention and very early control of severe viral, bacterial, fungal, and protozoal infections remains crucial for taming their progression into sepsis.

Other studies of the whole blood response to pediatric sepsis, reported over 1,000 upregulated and 1,401 downregulated genes that were expressed in blood cells based on genome-level expression profiling using the microarray technology (26, 27). The whole blood response to adult sepsis has identified two classes of genomic changes: “adaptive” and “inflammatory”. The former was dominated by T cells and natural killer (NK) cells, which displayed an upregulation of the T cell receptor signaling pathway and NK cell-mediated cytotoxicity. The “inflammatory” response displayed a preponderance of macrophages with upregulation of the Toll-like receptor signaling pathway linked to a less favorable prognosis (28). Our study of the two major organs, the lungs and kidneys, identified over 6,200 sepsis-responding genes in the lungs, and over 5,400 genes in the kidneys that were changed downward or upward during the 6-hour treatment with the selective nuclear blockade.

While comparing the genomic response to sepsis in the lungs and kidneys, we should consider structural and functional differences between them. Distinct regions of the lungs display different population of epithelial cells that function as adult stem cells. These cells are differentiated (59). Furthermore, the mature epithelium differs along the proximal-distal axis thereby determining its different potential for repair in response to injury (59). In turn, the air-blood barrier comprising alveolar-microvascular unit is particularly vulnerable to sepsis-causing microbial agents. They include viruses, such as influenza and SARS-Coronavirus 1 and 2, and LPS-bearing Gram-negative bacteria, the cause of sepsis in two-thirds of patients (11), as well as Staphylococci and Streptococci producing superantigenic immunotoxins (5, 15, 19).

The kidneys differ from the lungs in terms of their cell types and gene expression (60). In contrast to the lungs, the kidney cells are surrounded by the gradient of increasing tissue osmolality from the cortex to the medulla thereby influencing their transcriptomes. The kidney’s tubules traverse significant space from kidney’s cortex to medulla. This space is marked by steep corticomedullary gradients of tissue osmolality and oxygen tension, known to influence the gene expression of adjacent cells (61). In animal models of sepsis-caused AKI, cortical changes underlie the acute injury (25). Notwithstanding these spatial differences in the kidneys’ cellular and molecular architecture, the whole-organ gene expression profiling revealed that 33% of the 16,858 analyzed genes comprising the kidneys transcriptome were upregulated or downregulated in response to experimental sepsis (see above and **Figure 3**).

**Figure 5:**
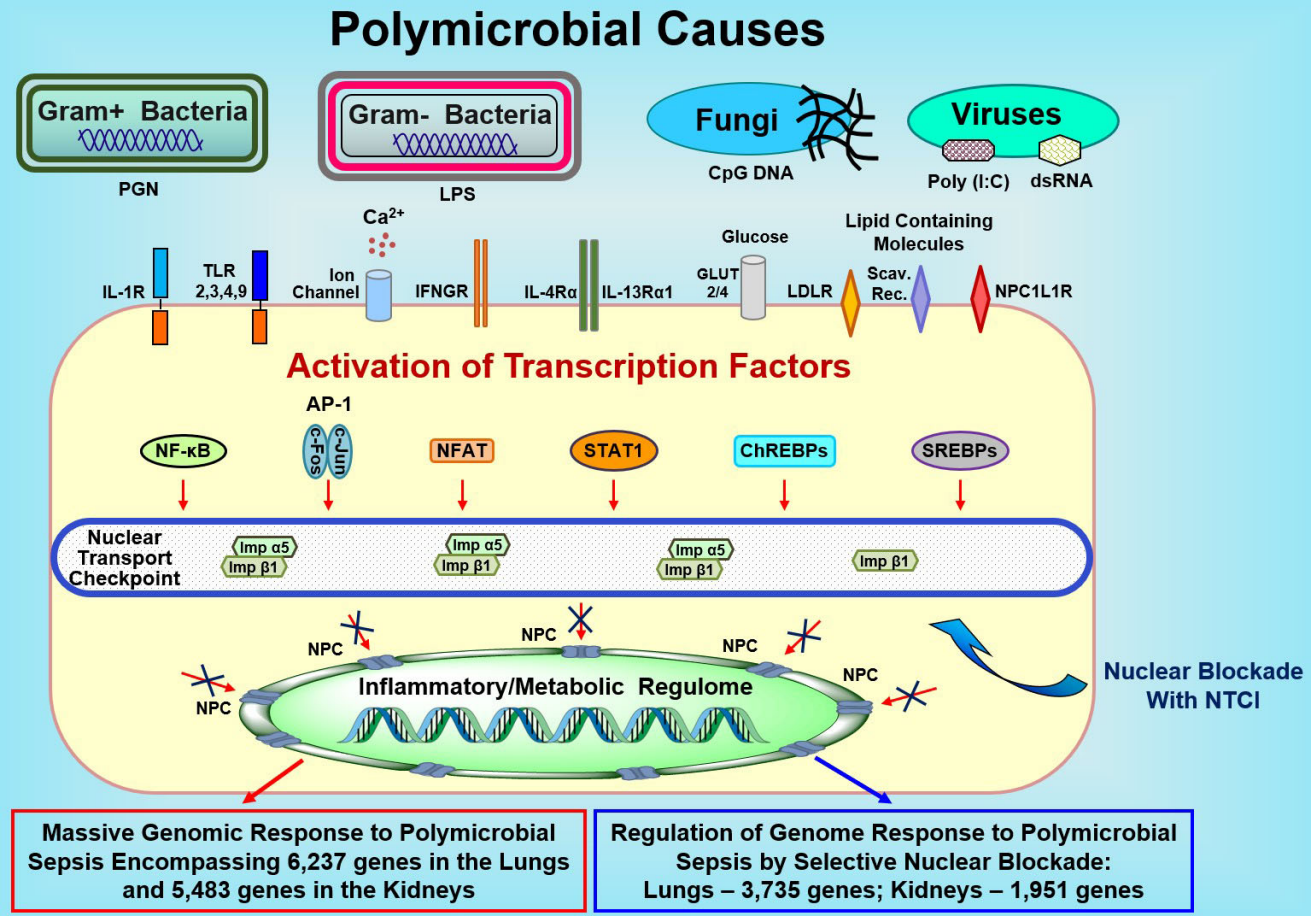
Sepsis-Causing Microbial Agents Sensed by Toll-Like Receptors and Other Extracellular and Intracellular Detectors Activate Multiple Transcription Factors in Immune and Non-Immune Cells (e.g., Endothelial and Epithelial Cells). Signals generated by these receptors/sensors/channels activate at least 7 families of stress-responsive transcription factors (SRTFs) and metabolic transcription factors (MTFs) Please note that the AP1 complex comprises two distinct transcription factors, cFos and cJun. Transcription factors are ferried to the inflammatory and/or metabolic regulomes in the nucleus by nuclear transport shuttles (importin α5 and importin β1) that are selectively controlled by the Nuclear Transport Checkpoint Inhibitors (NTCIs) (see text for details). Thus, selective nuclear blockade shields the inflammatory and metabolic regulomes in the nucleus thereby reducing the activation of a myriad (in thousands) of genes responding to sepsis. NPC, Nuclear Pore Complex.

The selective nuclear blockade imposed by the NTCI simultaneously targets two nuclear transport shuttles, cytoplasmic adaptor proteins termed importin α5 and importin β1 (16, 19, 20). These two cytoplasmic proteins are required for the nuclear translocation of an entire set of proinflammatory SRTFs and MTFs to the cell’s nucleus. Thus, the NTCI disables the inflammatory and metabolic regulomes thereby silencing (until this study) at least 32 known genes encoding the mediators of inflammation in animal models of allergic (62), autoimmune, metabolic, microbial, and physical inflammation (19). In the experimental sepsis model studied by us herein, the number of genes regulated by the selective nuclear blockade in the lungs and kidneys exceeds 3,700 and 1,900, respectively (see the conceptual figure, **Figure 5**).

The selectivity of the nuclear blockade imposed by disabling importin α5- and importin β1-based nuclear transport of SRTFs and MTFs allows other members of importins α family to continue their nuclear translocation function. Moreover, the potential adverse impact of inhibiting importin α5 was allied by the report that mice with an importin α5 deficiency are viable and fertile and do not display any apparent morphological and behavioral defects (63). Significantly, the NTCI inhibits the nuclear translocation of larger transcription factors (MW>45 kDa), such as SRTFs and MTFs by targeting the Imp α5/Imp β1 complex (16, 62). Therefore, in the presence of the NTCI, the smaller transcription factors (MW<45 kDa), which are essential to cell survival and maintenance, can freely translocate to the nucleus and contribute to the homeostasis and lifespan of cells treated with the NTCI (16). We found previously that the NTCI treatment did not alter the expression of the five housekeeping genes (*Gusb, Hprt1, Hsp90ab1, Gapdh*, and *Actb*) (17).

The new generation cell-penetrating NTCI, the cSN50.1 peptide, regulated the massive genomic response to sepsis and enhanced bacterial clearance without institution of antimicrobial therapy, as documented here. Previously, in the experimental model of sepsis, the same NTCI increased the survival of mice from 30% in the antibiotic-treated control group to 55% in the group treated with the NTCI combined with antimicrobial therapy (18). Another experimental sepsis study using a cecal ligation and puncture model, independently showed that the first generation NTCI (SN50 peptide), combined with antimicrobial therapy, also improved survival (64). Moreover, the innate immunity reprogramming action of the NTCI (SN50 peptide) was subsequently showed by others in the adjuvant-mediated enhancement of response to the viral and bacterial vaccines (65). Notably, a new generation NTCI (the cSN50.1 peptide) used herein is 8 times more water soluble and bioavailable than the first generation NTCI (SN50 peptide) used in most of the studies (17).

The NTCI reported in this and earlier reports, represents a new class of broad-spectrum anti-inflammatory agents for topical (localized) genomic control of inflammation (62) and, if needed, systemic therapy of sepsis and related diseases mediated by microbial inflammation (19). Other broad-spectrum anti-inflammatory agents, the glucocorticoids, e.g., dexamethasone, target the inflammatory regulome *via* the action of the cognate nuclear receptor, which functions as a transcription factor, inducing genomic response (66–68). Inadvertently, this response causes immunosuppression and dysregulated metabolism (hyperglycemia, hyperlipidemia) as well as skin atrophy, osteoporosis (19) and atherosclerosis (69). In striking contrast, the NTCI reduces blood glucose, cholesterol, and triglycerides, and atherosclerosis (16) while increasing the innate immunity-mediated clearance of bacteria in the blood, spleen, and lungs (**Figure 4**).

The microbial clearance, operational in the blood, spleen, lungs, and other organs, reduces the dissemination of bacteria from an initial site of sepsis-causing infection. This primarily phagocytic process, mediated by circulating myelomonocytic cells (granulocytes and monocytes) and tissue macrophages (56), also depends on well-functioning small blood vessels (<100 µm diameter). Their normal function in the lungs, heart, kidneys, and other organs is essential for trafficking and extravasation of phagocytic and non-phagocytic immune cells (5). Microbial agents, joined by the host-produced mediators of innate immunity and inflammation, damage the small blood vessels (5).

These considerations are important as the wide-spread emergence of microbes that can escape antimicrobial therapy calls for new approaches to cytoprotective treatments of severe infections evolving into sepsis. Among the cytoprotective approaches, pathogen-directed monoclonal antibodies tested in Ebola virus-associated sepsis (70) and other viral infections (71), recombinant SLIT protein (72), and NTCIs (19), await further preclinical and clinical studies. Frustratingly, over 100 published clinical trials in sepsis targeting microbial factors, cytokines (including anti-TNF-alpha and anti-IL-1 beta monoclonal antibodies), and blood coagulation regulators have thus far failed (73). A follow-up review analyzed more than 200 unique putative biomarkers of sepsis that have been proposed and concluded that none of these is used to guide therapies that target the host response (74).

The selective nuclear blockade that impedes the access of at least 7 families of transcription factors to the inflammatory and metabolic regulomes in the cell’s nucleus (**Figure 5**) controls cumulatively the expression of over 4,200 genes in the two major target organs in sepsis as documented herein. Moreover, it enhances the innate immunity-mediated bacterial clearance in the blood, spleen, and lungs (**Figure 4**) and increases survival when combined with anti-microbial therapy (18). These gains are imperative as sepsis-causing infections are increasingly difficult to control due to growing multi-drug resistance (5). For example, community-acquired Methicillin-Resistant Staphylococcus aureus (MRSA) almost doubled the number of influenza deaths (75).

The upregulated and downregulated genes in sepsis can be categorized in the following functional groups: A. The mediators of innate immunity and inflammation (cytokines, chemokines, growth factors, cell-adhesion molecules, intracellular signaling intermediates, microbicidal peptides). This set also encompasses genes responsible for the inborn errors of immunity based on the 320 different gene defects underlying 330 distinct disorders as reported in 2018 (76). B. The genes involved in the metabolism of cholesterol, triglycerides, fatty acids, and carbohydrates. C. The genes encoding transcription factors (19), including those participating in the feed-forward proinflammatory loop (e.g., cFOS), and metabolic signaling loops (e.g., SREBPs). D. The genes encoding physiologic suppressors of inflammation (e.g., SOCS1, SOCS3, CRADD, and A20) (19). Since microvascular endothelial cells are dysfunctional during sepsis (25), we emphasize a separate category (E) for the genes encoding the endothelial regulatory signaling axis comprising angiopoietin-1(angpt-1) and Tie2 and others (77). Microvascular endothelial injury plays a critically important role in the sepsis-induced dysfunction of the lungs, kidneys, and other organs.

Our genomic findings in experimental sepsis controlled by the selective nuclear blockade have significant translational implications for the mechanism of human sepsis and related complications. For example, the Intensive Care Unit-Acquired Weakness (ICUAW), that delays patients’ recovery is mediated by *Saa1* gene-encoded Serum Amyloid A1 contributing to the myotube injury (34). In addition, we identified in experimental sepsis another sepsis-suppressed gene linked to muscle dystrophy, anoctamin 5 (Ano5), and discovered its potentially beneficial regulation by the NTCI, Importantly, the treatment with the NTCI lowered the expression of Saa1 in the lungs by 2.7-fold. Furthermore, it lowered *Saa3* gene expression four-fold in the lungs and two-fold in the kidneys and raised 5-fold the expression of Anoctamin 5 gene (see **Suppl. Tables ST1A, ST2A**).

Disseminated intravascular coagulation (DIC), is one of the major hematologic complications of sepsis (5).The NTCI treatment reduced the expression of the genes encoding fibrinogen chains and Factor X in the lungs and kidneys potentially reducing the risk of DIC in sepsis.

Metabolic derangements in human sepsis exemplified by elevated lactate level in blood and alteration in fatty acid metabolism were increased in non-survivors of Septic Shock (78). Our study found strikingly profound changes in genes encoding endocrine and metabolic regulators. Hence, the G protein-coupled bile acid receptor-1 (*Gpbar1* aka Takeda G protein-coupled receptor-5 [TGR5]) was downregulated in experimental sepsis. TGR5 stimulates glucagon-like peptide-1 (GLP-1) secretion to improve insulin sensitivity and hepatic metabolism (54). Significantly, the treatment with the NTCI increased 4-fold the expression of the *Gpbar1*. This underscores the NTCI’s potential for metabolic improvement in sepsis and other severe infectious diseases. The two genes encoding cytochrome P450, family 11 subfamily b and a polypeptide 1, were most profoundly suppressed in experimental sepsis. The cytochrome P450 family 17 subfamily A member 1 participates in steroidogenesis induced by endothelin-1 (55). Sepsis-induced suppression of these genes heralds potential failure of physiologic steroidogenesis. The NTCI partially reversed precipitous decline of these metabolic genes. The last but not least example of potential translational value emerging from our study is the gene encoding β-Klotho. It was downregulated by sepsis in the lungs and kidneys and increased by the NTCI. The β-Klotho is the essential component of fibroblast growth factor (FGF) receptor complexes required for high-affinity binding of endocrine FGF19 and FGF21. They evoke the signaling cascade actively involved in homeostatic maintenance of glucose metabolism and energy expenditure that are dysregulated in sepsis (47).

In summary, we uncovered the hitherto unreported induction of thousands of genes by experimental sepsis in the lungs and kidneys, the two major organs, which are the sites of ARDS and AKI, respectively. We also found that this genomic response to sepsis is regulated by the selective nuclear blockade with the NTCI. The resulting reprogramming of the genes in the lungs, spleen, and blood contributes to the increased bacterial clearance reducing the dissemination of polymicrobial infection (see above **Figure 4**) and increasing survival in sepsis (18). Therefore, the selective nuclear blockade counteracts the action of the microbial virulence factors combined with the host’s produced mediators of inflammation thereby protecting the major organs from potentially lethal septic injury.

Altogether, our results are of significant relevance to millions of individuals in all age groups threatened by sepsis. We submit that the NTCI-based approach paves the way for the potentially effective treatment of sepsis, one of the most prevailing and challenging complication of severe infections worldwide (1). Our findings of robust genomic response to experimental sepsis and its successful control in the two major organs, lungs and kidneys, by the NTCI could be valuable for further medical use. The NTCI (cSN50.1 aka. AMTX-100 CF) is in the ongoing Phase 2 clinical trial for mild to moderate Atopic Dermatitis (NCT04313400), the most common skin disease (62).

## Data availability statement

All data generated or analyzed during this study is included in this published article and its supplementary information files. The NGS raw data is deposited in the NBCI Gene Expression Omnibus (GEO) repository of high throughput sequencing data. Accession number GSE239388.

## Ethics statement

The animal study was reviewed and approved by Vanderbilt University Institutional Animal Care and Use Committee.

## Author contributions

JZ and JH designed the study. HQ, YL, and JZ, performed experiments. HQ and JZ, acquired and analyzed data. JZ, and JH drafted the manuscript. JZ prepared figures. All authors reviewed the manuscript. All approved the submitted version.
