# Supplementary material for: Activation of thousands of genes in the lungs and kidneys by sepsis is countered by the selective nuclear blockade
Source: Front Immunol. 2023 Aug 11;14:1221102. doi: 10.3389/fimmu.2023.1221102 (PMC10450963; doi:10.3389/fimmu.2023.1221102)
Supplement: Supplementary file 1 [file DataSheet_1.pdf]

Supplemental Figure SF1

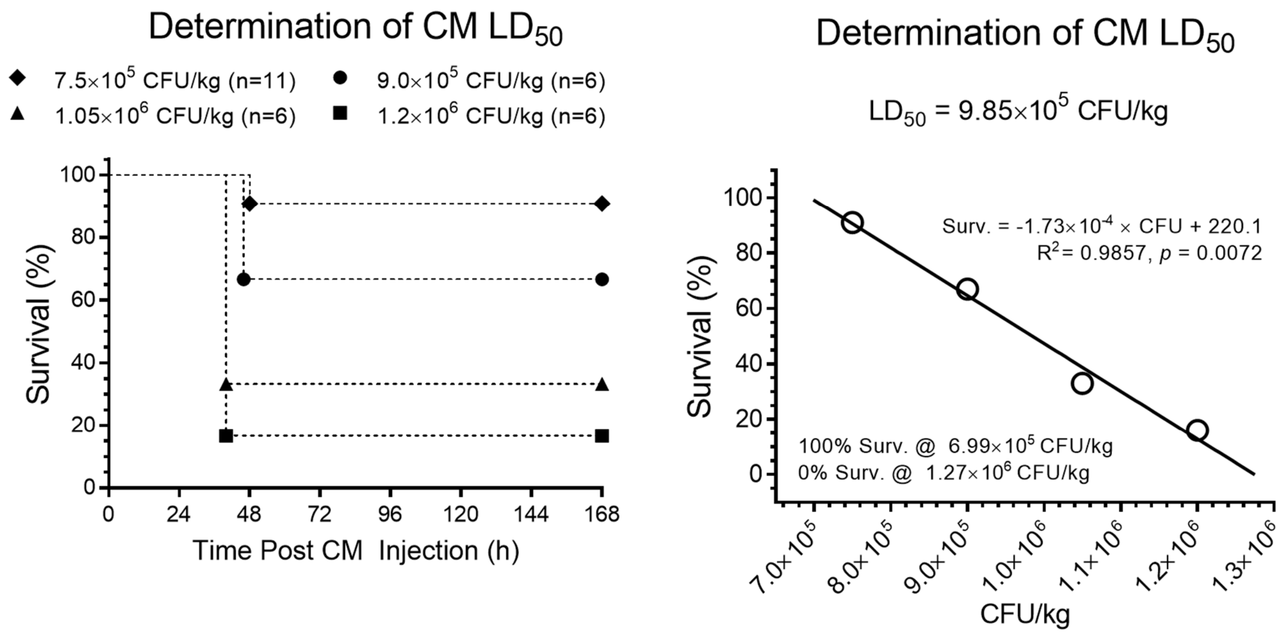

Suppl. Fig. SF1. Determination of Cecal Microbiome Lethal Dose Causing 50% Mortality. 8-week-old mice were challenged with a single i.p. injection of CM at a dose corresponding to the indicated CFUs. Mice were observed for 7 days after the CM-challenge without any treatment and the survival was recorded. Original observation is presented as the Kaplan–Meier survival plot (left panel). A dose- dependent survival represents a linear correlation according to the following equation: Survival = $-1.73\times10^4 \times$ CFU + 220.1 with R^2=0.9875 (p=0.0072). The LD_{50} corresponds to 9.85×10^5 CFU/kg of body mass.

Supplemental Figure SF2

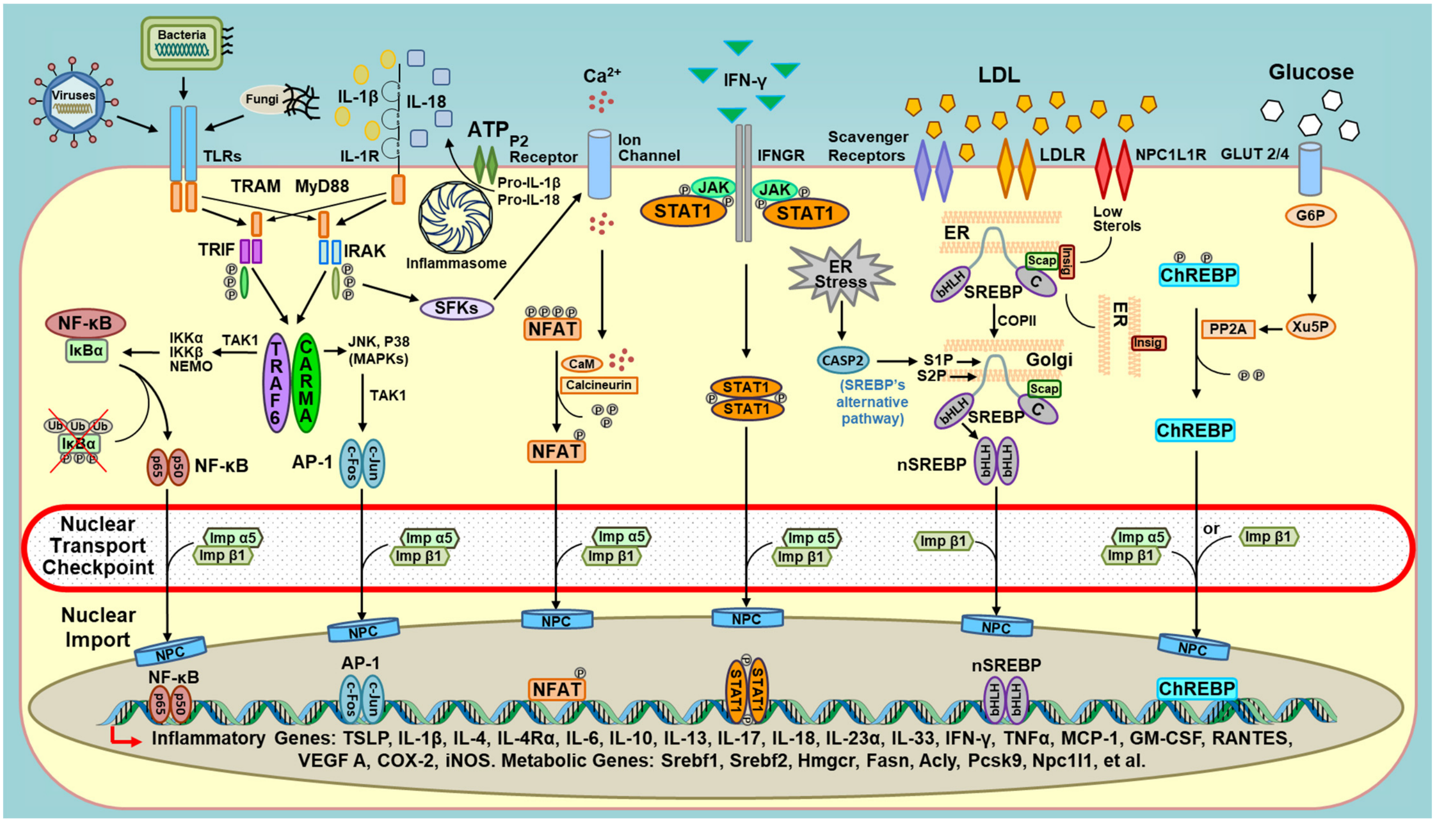

Suppl. Fig. S2. Signaling to the Nucleus by Inflammatory and Metabolic Transcriptional Pathways. In response to multiple proinflammatory cues outside of cell, the six selected transcriptional cascades, depicted schematically in this figure, are activated to trigger signaling to the genome in the cell's nucleus. This signaling is dependent on multiple receptors and their adapters, and other signal transducers (kinases, phosphatases, ubiquitin modifiers, and nuclear import adaptors) that form the signalosomes and other multimeric complexes. Proinflammatory Stress-Responsive Transcription Factors (SRTFs) encompass NF-κB, AP-1 (cFos, cJun), NFAT, and STAT1, among others. Metabolic Transcription Factors (MTFs) comprise Sterol Regulatory Element Binding Proteins (SREBPs) and Carbohydrate Regulatory Element Binding Proteins (ChREBPs), among others. Depicted SRTFs and MTFs converge at the Nuclear Transport Checkpoint "staffed" by Importin α5 (Imp α5) and importin β1 (Imp β1), among others. Imp α5 recognizes the Nuclear Localization Sequence (NLS) displayed in depicted SRTFs while Imp β1 recognizes the basic Helix-Loop-Helix leucine-rich zipper (bHLH-Zip) domain displayed in the SREBPs and ChREBPs. Moreover, Imp β1 recognizes its cognate binding site (importin beta binding domain) on Imp α5 ferrying its attached "payload" (i.e. transcription factor) to guide it to the Nuclear Pore Complex (NPC), and the genome. A nuclear form of SREBPs (nSREBPs) is ferried to the cell's nucleus solely by the Imp β1, which recognizes SREBP's N-terminal motif, bHLH-Zip. Other metabolic transcription factors, ChREBPs, display both bHLH-Zip and NLS. Once in the nucleus, SRTFs and MTFs bind to their cognate transcription factor binding sites in the gene promoters and enhancers to initiate transcription of the multiple genes that encode mediators of inflammatory and metabolic pathways. Some of these inflammatory and metabolic genes are displayed in the nucleus.

The interaction between "activated" or "processed" TFs and the nuclear transport shuttles can be inhibited by the cell-penetrating peptides termed Nuclear Transport Checkpoint Inhibitors aka. Nuclear Transport Modifiers (NTMs). Hence, the NTCIs aka NTMs dismantle the nuclear transport checkpoint by competitive binding to Imp α5 or Imp β1 thereby silencing the inflammatory regulome in the genome.

Supplemental Figure SF3

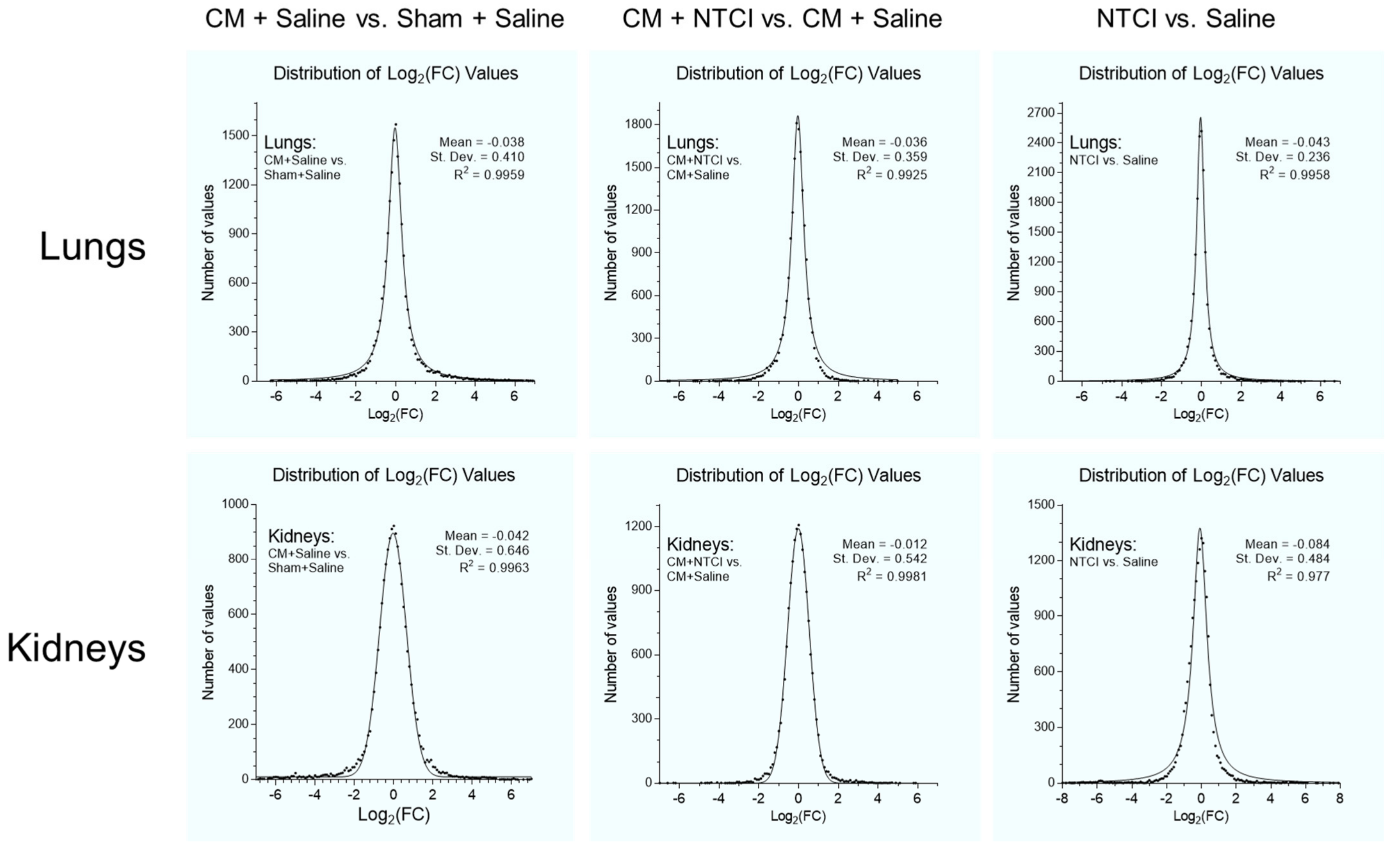

Suppl. Fig. S3. Distribution of the $\log_2$(FC) values. The Log_2(FC) values were rounded down to the nearest decimal point (0.1), and the equal values were added up. Data points were plotted against the $\log_2$(FC) to determine the center of distribution and the standard deviation used for selection of genes with increased, decreased, and unchanged expression. (See Materials and Methods for details).

Supplemental Figure SF4

SF4A

Kirby-Bauer Antimicrobial Susceptibility Test

CM: 200 µl of 4.2×10^3 CFU/ml

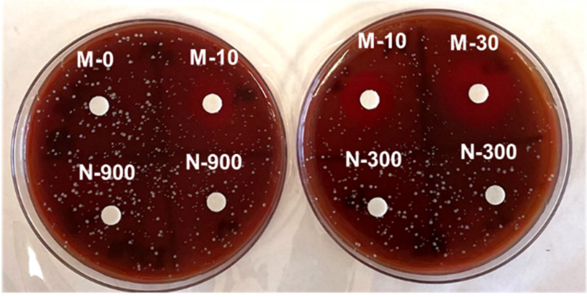

CM: 200 µl of 2.1×10^3 CFU/ml

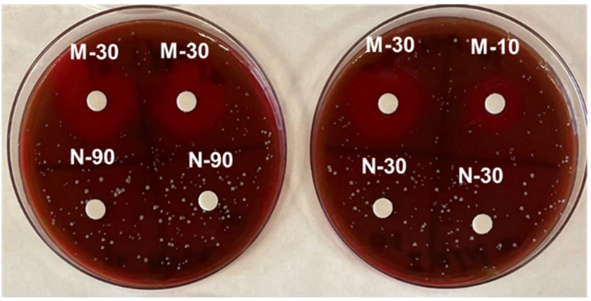

M - meropenem

M0 – 0 µg
M10 – 10 µg (26 nmol)
M30 – 30 µg (78 nmol)

N – NTCI

N30 – 30 µg (10 nmol)
N90 – 90 µg (30 nmol)
N300 – 300 µg (100 nmol
N900 – 900 µg (300 nmol)

SF4B

NTCI diffusion capability

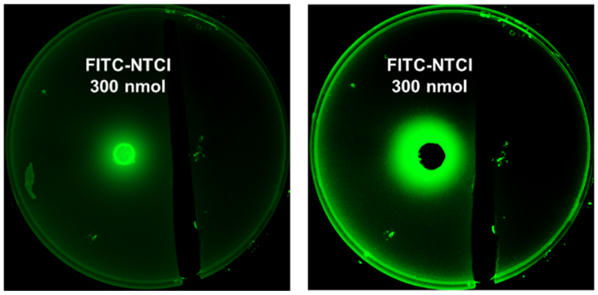

Suppl. Fig. SF4. Determination of Potential Direct Antibacterial Action of the NTCI. Suppl. Fig. SF4A. Potential NTCI's antibacterial action toward the constituents of the cecal microbiome (CM) was determined by the Kirby-Bauer's Antimicrobial Susceptibility Test (AST) with meropenem as a positive control. Sterile discs were loaded with the indicated amount of NTCI and placed on TSA + 5% sheep blood agar plates before application of the CM. The plates were incubated overnight at 37 °C. The Kirby-Bauer's AST indicates no effect of the NTCI on bacterial growth. **Suppl. Fig. SF4B**. The test of NTCI diffusion to the agar. The disc saturated with the FITC-labeled NTCI was placed on regular agar plate without CM, incubated for 12 hrs. at 37 °C, and visualized by the fluorescent imaging (left panel). To improve image quality by reducing strong signal emanating from non-diffused FITC-NTCI, which was accumulated on the disc and underneath, the disc and corresponding portion of the agar were removed from the plate (right panel). Please note that the portion of the agar gel was also removed from across the plate to avoid FITC-NTCI diffusion. The FITC-free area (right side of the plate) was then used as a basis for determining the background fluorescence.
