# Supplementary material for: Activation of thousands of genes in the lungs and kidneys by sepsis is countered by the selective nuclear blockade
Source: Front Immunol. 2023 Aug 11;14:1221102. doi: 10.3389/fimmu.2023.1221102 (PMC10450963; doi:10.3389/fimmu.2023.1221102)
Supplement: Supplementary file 2 [file DataSheet_2.pdf]

Supplemental Tables.

Genomic response to sepsis in the lungs and kidneys as illustrated by the heat map of the top 100 genes displaying the greatest gene expression fold change. ST1 A & B. Genes with increased and decreased expression in both organs (Shared Genes) sorted by the fold expression in the lungs. **ST2 A & B.** Top genes expressed in the lungs. **ST3 A & B.** Top genes expressed in the kidneys. The data in both conditions CM (CM + Saline) and NTCI (CM + cSN50.1) is presented as a fold expression compared to Sham + Saline.

Suppl. Tab. ST1A. Genes with Incresed Expression in Sepsis Shared by Lungs and Kidneys

			Lungs		Kidneys	
	Gene ID	Gene Name	Saline	NTCI	Saline	NTCI
1	*Csf3*	colony stimulating factor 3 (granulocyte)	1,347	1,296	847	1,168
2	*Il10*	interleukin 10	460	25	6.3	6.6
3	*Acod1*	aconitate decarboxylase 1	452	103	73	53
4	*Saa3*	serum amyloid A 3	412	106	23	12
5	*Cxcl2*	chemokine (C-X-C motif) ligand 2	323	215	217	555
6	*Gpr31b*	G protein-coupled receptor 31, D17Leh66b region	306	67	5.4	4.8
7	*Fgf23*	fibroblast growth factor 23	227	437	28	27
8	*Igfbp1*	insulin-like growth factor binding protein 1	111	80	4.6	8.0
9	*Mt2*	metallothionein 2	106	123	14	39
10	*Mrgpra2b*	MAS-related GPR, member A2B	103	78	29	71
11	*Ccl3*	chemokine (C-C motif) ligand 3	101	8.9	12	4.5
12	*Ccl4*	chemokine (C-C motif) ligand 4	98	2.9	12	3.0
13	*Gpr84*	G protein-coupled receptor 84	81	19	20	17
14	*Adamts4*	a disintegrin-like and metallopeptidase (reprolysin type) with thrombospondin type 1 motif, 4	78	86	27	16
15	*Madcam1*	mucosal vascular addressin cell adhesion molecule 1	76	46	47	20
16	*Il1r2*	interleukin 1 receptor, type II	73	145	7.8	32
17	*Olfm4*	olfactomedin 4	71	31	2.1	1.8
18	*Il1f9*	interleukin 36G	68	8.6	34	16
19	*Cxcl1*	chemokine (C-X-C motif) ligand 1	64	61	123	262
20	*Gpr31a*	G protein-coupled receptor 31, D17Leh66a region	62	16	12	3.8
21	*Gpr31c*	G protein-coupled receptor 31, D17Leh66c region	60	17	3.1	2.3
22	*Ptx3*	pentraxin related gene	57	47	137	227
23	*Slc7a11*	solute carrier family 7 (cationic amino acid transporter, y+ system), member 11	57	30	6.5	12
24	*Gm6553*	G protein-coupled receptor 31, D17Leh66c region pseudogene	55	17	14	9.8
25	*Tmprss11g*	transmembrane protease, serine 11g	54	103	18	39
26	*Tac1*	tachykinin 1	54	80	27	86
27	*Il1f6*	interleukin 36A	51	4.0	64	114
28	*Ccl2*	chemokine (C-C motif) ligand 2	50	16	16	17
29	*Psg16*	pregnancy specific beta-1-glycoprotein 16	48	39	9.7	6.0
30	*Fosl1*	fos-like antigen 1	48	31	11	24
31	*Sprr2a2*	small proline-rich protein 2A2	45	51	2.3	6.8
32	*Serpina3m*	serine (or cysteine) peptidase inhibitor, clade A, member 3M	45	29	17	7.4
33	*Il1rn*	interleukin 1 receptor antagonist	43	14	165	117
34	*Cxcl3*	chemokine (C-X-C motif) ligand 3	42	103	207	421
35	*Ccl20*	chemokine (C-C motif) ligand 20	41	41	8.1	8.1
36	*Tnf*	tumor necrosis factor	38	3.7	8.7	6.0
37	*Gm43181*	predicted gene 43181	37	13	23	15
38	*Clec4e*	C-type lectin domain family 4, member e	37	18	45	83
39	*Gm45774*	predicted gene 45774	35	22	7.3	3.8
40	*Ugt1a10*	UDP glycosyltransferase 1 family, polypeptide A10	35	39	3.5	1.9
41	*Kcnt1*	potassium channel, subfamily T, member 1	32	28	2.4	2.1
42	*Mt1*	metallothionein 1	31	39	5.9	19
43	*Mefv*	Mediterranean fever	31	20	17	18
44	*Clec4d*	C-type lectin domain family 4, member d	31	16	12	13
45	*Ms4a4a*	membrane-spanning 4-domains, subfamily A, member 4A	31	12	12	7.1
46	*Btbd16*	BTB (POZ) domain containing 16	30	10	32	18
47	*Spic*	Spi-C transcription factor (Spi-1/PU.1 related)	29	13	3.1	4.0
48	*Orm1*	orosomucoid 1	29	10	7.8	2.6
49	*Serpina3f*	serine (or cysteine) peptidase inhibitor, clade A, member 3F	28	15	76	86
50	*Il1b*	interleukin 1 beta	27	16	4.7	4.7
51	*Serpina10*	serine (or cysteine) peptidase inhibitor, clade A (alpha-1 antiproteinase, antitrypsin), member 10	27	9.3	6.4	2.1
52	*9830107B12Rik*	RIKEN cDNA 9830107B12 gene	27	9.3	22	4.5
53	*Gm49839*	predicted gene, 49839	27	43	10.0	16
54	*Msr1*	macrophage scavenger receptor 1	27	7.2	6.0	5.5
55	*Ankrd2*	ankyrin repeat domain 2 (stretch responsive muscle)	26	14	13	15
56	*Saa4*	serum amyloid A 4	25	5.5	14	0.8
57	*S100a8*	S100 calcium binding protein A8 (calgranulin A)	25	14	15	21
58	*Hpx*	hemopexin	24	6.6	25	1.3
59	*Apoa4*	apolipoprotein A-IV	24	2.2	1.8	2.2
60	*Steap4*	STEAP family member 4	23	15	39	17
61	*Cxcl10*	chemokine (C-X-C motif) ligand 10	22	12	9.3	7.5
62	*S100a9*	S100 calcium binding protein A9 (calgranulin B)	22	15	14	21
63	*Wfdc17*	WAP four-disulfide core domain 17	22	5.1	3.5	3.2
64	*Mmp8*	matrix metallopeptidase 8	22	19	233	282
65	*Gnat1*	G protein subunit alpha transducin 1	22	17	5.7	19

66	*Fgg*	fibrinogen gamma chain	21	7.3	36	19
67	*Fgb*	fibrinogen beta chain	20	6.2	26	2.3
68	*Lilr4b*	leukocyte immunoglobulin-like receptor, subfamily B, member 4B	20	13	3.8	6.2
69	*2010003K11Rik*	RIKEN cDNA 2010003K11 gene	20	5.8	5.8	9.5
70	*Serpina3n*	serine (or cysteine) peptidase inhibitor, clade A, member 3N	20	11	29	9.3
71	*AA467197*	expressed sequence AA467197	19	14	39	40
72	*Gm16685*	predicted gene, 16685	19	19	4.1	3.2
73	*Timp1*	tissue inhibitor of metalloproteinase 1	19	14	6.7	5.6
74	*Fga*	fibrinogen alpha chain	18	5.5	37	24
75	*Ifitm6*	interferon induced transmembrane protein 6	18	19	7.0	14
76	*Lcn2*	lipocalin 2	18	9.5	53	53
77	*F10*	coagulation factor X	18	3.3	4.9	1.2
78	*Leap2*	liver-expressed antimicrobial peptide 2	17	4.3	6.4	0.5
79	*Il6*	interleukin 6	17	56	223	671
80	*Retnlg*	resistin like gamma	16	16	9.1	22
81	*Ccl7*	chemokine (C-C motif) ligand 7	16	6.6	13	19
82	*Ikbke*	inhibitor of kappaB kinase epsilon	15	3.9	2.9	1.4
83	*Vcan*	versican	14	8.1	6.8	9.0
84	*F13a1*	coagulation factor XIII, A1 subunit	14	5.9	2.5	1.8
85	*Serpina3g*	serine (or cysteine) peptidase inhibitor, clade A, member 3G	14	9.3	14	11
86	*1200007C13Rik*	RIKEN cDNA 1200007C13 gene	14	19	35	73
87	*Ngp*	neutrophilic granule protein	13	13	481	612
88	*Siglece*	sialic acid binding Ig-like lectin E	13	5.2	3.1	2.1
89	*Lrrc25*	leucine rich repeat containing 25	13	5.8	3.1	2.7
90	*Gm10309*	predicted gene 10309	13	8.4	36	20
91	*Trem1*	triggering receptor expressed on myeloid cells 1	13	9.3	9.0	15
92	*Slc39a14*	solute carrier family 39 (zinc transporter), member 14	13	8.4	4.6	4.3
93	*Fgl1*	fibrinogen-like protein 1	13	4.3	3.5	1.0
94	*Il27*	interleukin 27	12	2.5	8.5	6.0
95	*Tcp10b*	t-complex protein 10b	12	13	4.7	2.7
96	*Serpina3i*	serine (or cysteine) peptidase inhibitor, clade A, member 3I	12	11	26	68
97	*Cxcl9*	chemokine (C-X-C motif) ligand 9	12	3.7	16	16
98	*A730049H05Rik*	RIKEN cDNA A730049H05 gene	12	30	2.4	2.3
99	*Gm8883*	predicted gene 8883	12	0.7	3.7	1.7
100	*Fpr2*	formyl peptide receptor 2	12	8.5	25	31

Suppl. Tab. ST1B. Genes with Decreased Expression in Sepsis Shared by Lungs and Kidneys

			Lungs		Kidneys	
	Gene ID	Gene Name	Saline	NTCI	Saline	NTCI
1	*Ano5*	anoctamin 5	-53	-9.3	-17	-11
2	*Gsg1l*	GSG1-like	-31	-6.9	-72	-126
3	*Spon2*	spondin 2, extracellular matrix protein	-27	-13	-16	-2.9
4	*Oxtr*	oxytocin receptor	-26	-1.0	-22	-14
5	*Rnf112*	ring finger protein 112	-21	-12	-39	NS
6	*Ppp2r2c*	protein phosphatase 2, regulatory subunit B, gamma	-17	-5.1	-4.3	-33
7	*Crabp1*	cellular retinoic acid binding protein I	-16	-7.5	-30	-4.8
8	*St6galnac3*	ST6 (alpha-N-acetyl-neuraminyl-2,3-beta-galactosyl-1,3)-N-acetylgalactosaminide alpha-2,6-sialyltransferase 3	-15	-6.7	-4.1	-3.1
9	*Agtr2*	angiotensin II receptor, type 2	-12	-1.5	-23	-39
10	*Gjc2*	gap junction protein, gamma 2	-11	-9.4	-23	-28
11	*Gm33195*	predicted gene, 33195	-11	-1.3	-5.8	-2.0
12	*Gabra4*	gamma-aminobutyric acid (GABA) A receptor, subunit alpha 4	-10	-5.0	-3.8	-5.1
13	*Aplnr*	apelin receptor	-10	-4.0	-3.2	-5.7
14	*E030013I19Rik*	RIKEN cDNA E030013I19 gene	-9.2	-6.9	-6.1	-3.0
15	*Pcdh12*	protocadherin 12	-8.9	-10	-3.2	-4.1
16	*Chp2*	calcineurin-like EF hand protein 2	-8.6	-5.5	-4.2	-2.7
17	*Gng8*	guanine nucleotide binding protein (G protein), gamma 8	-8.6	-4.5	-20	-48
18	*Pla2g2d*	phospholipase A2, group IID	-8.1	-3.0	-4.7	-3.5
19	*Gm20683*	predicted gene 20683	-7.9	-1.5	-2.0	-1.2
20	*Mansc4*	MANSC domain containing 4	-7.7	-4.4	-2.7	-1.8
21	*Gm10824*	predicted gene 10824	-7.5	-4.6	-5.3	-5.1
22	*Ces2b*	carboxyesterase 2B	-7.5	-4.6	-2.5	-1.4
23	*Kank4*	KN motif and ankyrin repeat domains 4	-7.4	-12	-2.7	-4.2
24	*Zmat4*	zinc finger, matrin type 4	-7.4	-9.5	-3.9	-2.1
25	*Cdr1os*	cerebellar degeneration related antigen 1, opposite strand	-7.2	-3.5	-43	NS
26	*Wnt2*	wingless-type MMTV integration site family, member 2	-7.1	-6.0	-4.0	-2.5
27	*Gm17040*	predicted gene 17040	-6.7	-1.4	-2.9	-1.7
28	*Wfdc16*	WAP four-disulfide core domain 16	-6.7	-4.7	-2.2	-1.0
29	*Rab42*	RAB42, member RAS oncogene family	-6.7	-5.6	-5.0	-7.9
30	*Tbxa2r*	thromboxane A2 receptor	-6.7	-8.8	-4.4	-9.7
31	*Plppr5*	phospholipid phosphatase related 5	-6.6	-2.4	-47	-6.2
32	*Misp3*	MISP family member 3	-6.3	-11	-2.6	-1.7
33	*Mmp28*	matrix metallopeptidase 28 (epilysin)	-6.3	-6.4	-1.8	-3.2
34	*Lrrn4*	leucine rich repeat neuronal 4	-6.2	-3.6	-11	-6.1
35	*Sost*	sclerostin	-6.1	-8.5	-107	-17
36	*Tmem200a*	transmembrane protein 200A	-6.1	-4.8	-10	-5.6
37	*Shisa2*	shisa family member 2	-6.1	-11	-1.9	-2.6
38	*Stmn2*	stathmin-like 2	-5.9	-2.4	-3.8	-2.5
39	*Chrm1*	cholinergic receptor, muscarinic 1, CNS	-5.9	-3.0	-6.6	-25
40	*Tmeff2*	transmembrane protein with EGF-like and two follistatin-like domains 2	-5.9	-2.8	-7.7	-4.9
41	*2810433D01Rik*	RIKEN cDNA 2810433D01 gene	-5.8	-7.0	-3.6	-2.6
42	*Pabpc4l*	poly(A) binding protein, cytoplasmic 4-like	-5.8	-5.9	-6.4	-18
43	*Dact3*	dishevelled-binding antagonist of beta-catenin 3	-5.7	-6.0	-4.9	-7.4
44	*Mettl24*	methyltransferase like 24	-5.7	-2.7	-3.2	-1.5
45	*Gpr34*	G protein-coupled receptor 34	-5.7	-4.7	-14	-8.3
46	*D430019H16Rik*	RIKEN cDNA D430019H16 gene	-5.6	-8.9	-6.6	-13
47	*Hoxc5*	homeobox C5	-5.5	-13	-2.1	-3.7
48	*Tekt5*	tektin 5	-5.4	-2.2	-5.9	-6.1
49	*Megf10*	multiple EGF-like-domains 10	-5.4	-2.5	-34	-22
50	*Wscd1*	WSC domain containing 1	-5.4	-4.2	-2.5	-4.1
51	*Gm32219*	predicted gene, 32219	-5.4	-2.8	-51	-33
52	*Tril*	TLR4 interactor with leucine-rich repeats	-5.3	-5.2	-3.5	-5.3
53	*Agtr1b*	angiotensin II receptor, type 1b	-5.3	-2.6	-17	-41
54	*2610203C22Rik*	RIKEN cDNA 2610203C22 gene	-5.2	-6.0	-4.1	-4.5
55	*Abcg3*	ATP binding cassette subfamily G member 3	-5.2	-6.9	-13	-9.5
56	*Star*	steroidogenic acute regulatory protein	-5.2	-1.4	-914	-588
57	*Grik2*	glutamate receptor, ionotropic, kainate 2 (beta 2)	-5.1	-2.1	-39	-6.1
58	*Grin2c*	glutamate receptor, ionotropic, NMDA2C (epsilon 3)	-5.1	-1.7	-5.3	-2.8
59	*Vstm5*	V-set and transmembrane domain containing 5	-5.1	-3.8	-2.7	-2.2
60	*Smad9*	SMAD family member 9	-5.1	-6.1	-4.4	-6.3
61	*Pianp*	PILR alpha associated neural protein	-4.9	-3.7	-12	-5.4
62	*Rnf144a*	ring finger protein 144A	-4.9	-5.3	-1.7	-1.6
63	*Cthrc1*	collagen triple helix repeat containing 1	-4.8	-5.7	-16	-3.4
64	*Nxpe4*	neurexophilin and PC-esterase domain family, member 4	-4.8	-3.9	-1.8	-2.0
65	*Mrc2*	mannose receptor, C type 2	-4.8	-2.8	-2.3	-2.1
66	*Cx3cr1*	chemokine (C-X3-C motif) receptor 1	-4.8	-8.8	-4.8	-13

67	*Il5ra*	interleukin 5 receptor, alpha	-4.8	-2.8	-4.2	-8.0
68	*Kcnc4*	potassium voltage gated channel, Shaw-related subfamily, member 4	-4.7	-1.9	-5.4	-2.9
69	*Fam163a*	family with sequence similarity 163, member A	-4.7	-18	-2.1	-1.8
70	*Cxcr3*	chemokine (C-X-C motif) receptor 3	-4.6	-3.2	-3.3	-3.7
71	*A330074K22Rik*	RIKEN cDNA A330074K22 gene	-4.6	-12	-75	-25
72	*Gm11627*	predicted gene 11627	-4.6	-3.2	-13	-7.3
73	*Mmp11*	matrix metallopeptidase 11	-4.6	-5.9	-1.9	-1.2
74	*Fam181b*	family with sequence similarity 181, member B	-4.6	-4.9	-3.3	-5.3
75	*Cd209b*	CD209b antigen	-4.5	-2.2	-53	-4.9
76	*Tnfrsf19*	tumor necrosis factor receptor superfamily, member 19	-4.5	-3.6	-27	-19
77	*Sept4*	septin 4	-4.5	-3.4	-3.7	-2.7
78	*Nlgn3*	neuroligin 3	-4.4	-3.0	-173	-426
79	*Baalc*	brain and acute leukemia, cytoplasmic	-4.4	-2.0	-8.9	-4.5
80	*Ngb*	neuroglobin	-4.4	-3.7	-10	-4.0
81	*Nxph3*	neurexophilin 3	-4.4	-3.6	-8.6	-2.1
82	*Klb*	klotho beta	-4.3	-2.5	-5.1	-2.7
83	*Igkv4-72*	immunoglobulin kappa chain variable 4-72	-4.3	-7.2	-31	-20
84	*Ptprv*	protein tyrosine phosphatase receptor type V	-4.3	-2.6	-30	-2.3
85	*Nrep*	neuronal regeneration related protein	-4.2	-1.7	-2.6	-9.4
86	*Itga8*	integrin alpha 8	-4.2	-1.8	-2.9	-1.4
87	*Myct1*	myc target 1	-4.2	-8.1	-4.9	-10.0
88	*Palm*	paralemmin	-4.2	-4.3	-2.2	-2.2
89	*Syt3*	synaptotagmin III	-4.2	-1.9	-1.9	-1.1
90	*Tspan18*	tetraspanin 18	-4.2	-1.8	-2.3	-3.0
91	*Piezo2*	piezo-type mechanosensitive ion channel component 2	-4.1	-2.7	-3.2	-0.9
92	*Nat8f3*	N-acetyltransferase 8 (GCN5-related) family member 3	-4.1	-8.9	-1.7	-2.9
93	*Cngb1*	cyclic nucleotide gated channel beta 1	-4.1	-1.2	-8.8	-1.7
94	*Cd209g*	CD209g antigen	-4.1	-19	-32	-4.1
95	*Faim2*	Fas apoptotic inhibitory molecule 2	-4.0	-3.4	-5.5	-13
96	*Gfra2*	glial cell line derived neurotrophic factor family receptor alpha 2	-4.0	-3.2	-4.7	-4.1
97	*Srpk3*	serine/arginine-rich protein specific kinase 3	-4.0	-3.1	-74	-3.3
98	*Gm3716*	predicted gene 3716	-4.0	-4.2	-7.1	-6.2
99	*Klhl13*	kelch-like 13	-4.0	-3.1	-2.6	-1.5
100	*Mogat2*	monoacylglycerol O-acyltransferase 2	-3.9	-6.1	-2.4	-1.2

Suppl. Tab. ST2A. Genes with Inceased Expression in Lungs of Infected Mice

			Lungs	
	Gene ID	Gene Name	Saline	NTCI
1	*Saa2*	serum amyloid A 2	3,985	1,338
2	*Saa1*	serum amyloid A 1	1,849	658
3	*Prok2*	prokineticin 2	1,783	450
4	*B230303A05Rik*	U1 small nuclear ribonucleoprotein 1C pseudogene	1,612	697
5	*Gm43079*	predicted gene 43079	423	1.0
6	*Gm49383*	predicted gene, 49383	309	167
7	*Cldn14*	claudin 14	270	119
8	*Gm15056*	predicted gene 15056	200	17
9	*U90926*	cDNA sequence U90926	190	9.9
10	*Stfa2*	stefin A2	185	90
11	*Prokr2*	prokineticin receptor 2	171	4.5
12	*Lbx1*	ladybird homeobox 1	167	105
13	*Cstdc5*	cystatin domain containing 5	123	101
14	*Tarm1*	T cell-interacting, activating receptor on myeloid cells 1	119	41
15	*Mrgpra2a*	MAS-related GPR, member A2A	88	64
16	*Cstdc4*	cystatin domain containing 4	88	80
17	*Gm48786*	predicted gene, 48786	84	32
18	*Gm19510*	predicted gene, 19510	84	4.3
19	*Gm32089*	predicted gene, 32089	74	29
20	*Gm5966*	predicted gene 5966	67	30
21	*Stfa3*	stefin A3	63	39
22	*Tmem132e*	transmembrane protein 132E	58	87
23	*Orm2*	orosomucoid 2	56	12
24	*Gm28551*	predicted gene 28551	56	63
25	*A630023A22Rik*	RIKEN cDNA A630023A22 gene	55	50
26	*Gm7457*	predicted gene 7457	54	43
27	*Serpina7*	serine (or cysteine) peptidase inhibitor, clade A (alpha-1 antiproteinase, antitrypsin), member 7	53	25
28	*Bmerb1*	bMERB domain containing 1	49	28
29	*Klk9*	kallikrein related-peptidase 9	48	29
30	*1700011B04Rik*	RIKEN cDNA 1700011B04 gene	48	15
31	*Asprv1*	aspartic peptidase, retroviral-like 1	48	12
32	*Dmbt1*	deleted in malignant brain tumors 1	48	118
33	*Tmprss11d*	transmembrane protease, serine 11d	47	7.4
34	*Slc5a7*	solute carrier family 5 (choline transporter), member 7	43	18
35	*Ifitm7*	interferon induced transmembrane protein 7	42	17
36	*Gm38340*	predicted gene, 38340	41	26
37	*Pglyrp3*	peptidoglycan recognition protein 3	41	22
38	*Gm26748*	predicted gene, 26748	40	13
39	*4930431P03Rik*	RIKEN cDNA 4930431P03 gene	39	11
40	*4732490B19Rik*	RIKEN cDNA 4732490B19 gene	38	134
41	*Sectm1b*	secreted and transmembrane 1B	37	38
42	*Gm4948*	trans-2,3-enoyl-CoA reductase pseudogene	36	229
43	*Gm20406*	predicted gene, 20406	33	5.2
44	*Rasl10b*	RAS-like, family 10, member B	33	13
45	*Gm36079*	annexin A2 receptor 2	32	12
46	*Sult2a3*	sulfotransferase family 2A, dehydroepiandrosterone (DHEA)-preferring, member 3	32	3.6
47	*Apcs*	amyloid P component, serum	32	11
48	*Mymk*	myomaker, myoblast fusion factor	31	11
49	*Nkx2-9*	NK2 homeobox 9	29	3.8
50	*Gm26535*	predicted gene, 26535	29	7.5
51	*Rnase2a*	ribonuclease, RNase A family, 2A (liver, eosinophil-derived neurotoxin)	29	5.9
52	*Slc22a20*	solute carrier family 22 (organic anion transporter), member 20	28	6.0
53	*Stfa2l1*	stefin A2 like 1	28	24
54	*Gm4949*	glutamate-ammonia ligase (glutamine synthetase) pseudogene	28	81
55	*Gm11738*	predicted gene 11738	27	556
56	*Gm29686*	predicted gene, 29686	27	9.3
57	*Serpine3*	serpin peptidase inhibitor, clade E (nexin, plasminogen activator inhibitor type 1), member 3	27	9.9
58	*Gm807*	predicted gene 807	26	45
59	*Gm26444*	predicted gene, 26444	26	16
60	*Trdv5*	T cell receptor delta variable 5	25	4.3
61	*Sult1e1*	sulfotransferase family 1E, member 1	25	8.6
62	*Serpina3e-ps*	serine (or cysteine) peptidase inhibitor, clade A, member 3E, pseudogene	25	16
63	*4930578G10Rik*	RIKEN cDNA 4930578G10 gene	24	22
64	*Gm37645*	predicted gene, 37645	24	3.6
65	*Ceacam19*	CEA cell adhesion molecule 19	23	3.2

66	*Ackr1*	atypical chemokine receptor 1 (Duffy blood group)	23	12
67	*Ms4a3*	membrane-spanning 4-domains, subfamily A, member 3	23	1.9
68	*B130046B21Rik*	RIKEN cDNA B130046B21 gene	23	30
69	*Ctcflos*	CCCTC-binding factor (zinc finger protein)-like, opposite strand	23	17
70	*Doc2b*	double C2, beta	23	24
71	*Gm49573*	predicted gene, 49573	22	1.0
72	*Gm38275*	predicted gene, 38275	21	6.5
73	*Tex44*	testis expressed 44	21	2.1
74	*Gpr22*	G protein-coupled receptor 22	21	11
75	*Ceacam10*	CEA cell adhesion molecule 10	21	16
76	*E030018B13Rik*	RIKEN cDNA E030018B13 gene	20	6.0
77	*Gm37131*	predicted gene, 37131	20	14
78	*Htr3a*	5-hydroxytryptamine (serotonin) receptor 3A	20	8.8
79	*Slc13a5*	solute carrier family 13 (sodium-dependent citrate transporter), member 5	20	6.6
80	*Nxf3*	nuclear RNA export factor 3	19	6.6
81	*Gm49427*	predicted gene, 49427	19	3.2
82	*Pde6h*	phosphodiesterase 6H, cGMP-specific, cone, gamma	19	10
83	*Ubd*	ubiquitin D	19	5.5
84	*Fcrlb*	Fc receptor-like B	19	7.5
85	*Sult2a5*	sulfotransferase family 2A, dehydroepiandrosterone (DHEA)-preferring, member 5	19	4.1
86	*Tat*	tyrosine aminotransferase	18	11
87	*Gm6610*	predicted gene 6610	18	-1.9
88	*Gm44292*	predicted gene, 44292	18	21
89	*Gm44165*	predicted gene, 44165	17	12
90	*Prss22*	protease, serine 22	17	41
91	*Gm11714*	predicted gene 11714	16	32
92	*Gm49368*	predicted gene, 49368	16	2.6
93	*Gm48065*	predicted gene, 48065	16	23
94	*Aoah*	acyloxyacyl hydrolase	16	5.5
95	*Tctex1d1*	dynein light chain Tctex-type 5	16	2.7
96	*Phox2a*	paired-like homeobox 2a	16	19
97	*Slc36a3*	solute carrier family 36 (proton/amino acid symporter), member 3	15	5.7
98	*Sult2a1*	sulfotransferase family 2A, dehydroepiandrosterone (DHEA)-preferring, member 1	15	3.2
99	*Gm37347*	predicted gene, 37347	15	6.9
100	*Gm50083*	predicted gene, 50083	15	3.7

Suppl. Tab. ST3B. Genes with Decreased Expression in Lungs of Infected Mice

			Lungs	
	Gene ID	Gene Name	Saline	NTCI
1	*Tmem233*	transmembrane protein 233	-77	NS
2	*Duxbl3*	double homeobox B-like 1	-71	-2.2
3	*Rnase1*	ribonuclease, RNase A family, 1 (pancreatic)	-58	NS
4	*Defb42*	defensin beta 42	-57	NS
5	*AL935121.1*	predicted gene, 52968	-54	-2.2
6	*Zp1*	zona pellucida glycoprotein 1	-53	-14
7	*Snurf*	SNRPN upstream reading frame	-47	NS
8	*Gm25541*	predicted gene, 25541	-45	-19
9	*4933403O08Rik*	RIKEN cDNA 4933403O08 gene aka. testis expressed gene 16 (Tex16)	-40	NS
10	*1810046K07Rik*	POU domain, class 2, associating factor 2	-40	-7.1
11	*Gm5639*	PWWP domain containing 4, pseudogene	-38	-3.2
12	*Myh2*	myosin, heavy polypeptide 2, skeletal muscle, adult	-35	-5.0
13	*Gm765*	MyoD family inhibitor domain containing 2	-35	-6.2
14	*Gm50242*	predicted gene, 50242	-34	-9.0
15	*Gm6018*	MAD1 mitotic arrest deficient 1-like 1 pseudogene	-33	NS
16	*Ackr4*	atypical chemokine receptor 4	-28	-17
17	*Tnfrsf17*	tumor necrosis factor receptor superfamily, member 17	-28	-1.1
18	*Gm37858*	predicted gene, 37858	-28	-16
19	*Igkv14-130*	immunoglobulin kappa variable 14-130	-27	-364
20	*Ptprh*	protein tyrosine phosphatase receptor type H	-26	-14
21	*Gpr165*	G protein-coupled receptor 165	-25	NS
22	*Gm47652*	predicted gene, 47652	-25	-4.9
23	*Gm16534*	family with sequence similarity 78, member A	-23	-1.4
24	*0610012D04Rik*	RIKEN cDNA 0610012D04 gene	-23	-13
25	*Gm36569*	predicted gene, 36569	-23	-5.2
26	*Gzmk*	granzyme K	-23	-8.1
27	*Gm9918*	predicted gene 9918	-21	-1.8
28	*Gm33677*	predicted gene, 33677	-19	-1.3
29	*St6gal2*	beta galactoside alpha 2,6 sialyltransferase 2	-19	-5.7
30	*Krtap17-1*	keratin associated protein 17-1	-18	-2.5
31	*Olfr1342*	olfactory receptor family 13 subfamily P member 4	-17	-136
32	*Lhcgr*	luteinizing hormone/choriogonadotropin receptor	-17	-5.8
33	*Ccdc42*	coiled-coil domain containing 42	-16	-8.6
34	*Zfp985*	zinc finger protein 985	-16	-4.5
35	*Igkv4-70*	immunoglobulin kappa chain variable 4-70	-16	-14
36	*Gm31517*	predicted gene, 31517	-15	-5.3
37	*Gm9767*	predicted gene 9767	-14	-1.2
38	*Olfr71*	olfactory receptor family 13 subfamily J member 1	-14	-13
39	*Slc26a3*	solute carrier family 26, member 3	-13	-9.7
40	*Trpv3*	transient receptor potential cation channel, subfamily V, member 3	-13	-3.6
41	*Gm49727*	predicted gene, 49727	-13	-8.4
42	*Gm43951*	RIKEN cDNA 4933427D14 gene	-13	-2.0
43	*E330018M18Rik*	RIKEN cDNA E330018M18 gene	-13	-9.8
44	*Calcb*	calcitonin-related polypeptide, beta	-13	-3.1
45	*Rspo2*	R-spondin 2	-12	-18
46	*Glp1r*	glucagon-like peptide 1 receptor	-12	-13
47	*Fam124b*	family with sequence similarity 124, member B	-12	-10
48	*Amtn*	amelotin	-12	-3.9
49	*4930570G19Rik*	RIKEN cDNA 4930570G19 gene	-12	-2.9
50	*Gm11574*	predicted gene 11574	-12	-4.5
51	*Pla2g2e*	phospholipase A2, group IIE	-11	-1.1
52	*Sfrp2*	secreted frizzled-related protein 2	-11	-6.5
53	*Pigz*	phosphatidylinositol glycan anchor biosynthesis, class Z	-11	-6.5
54	*Gm13688*	predicted gene 13688	-11	-3.5
55	*Abca14*	ATP-binding cassette, sub-family A (ABC1), member 14	-11	-1.0
56	*B530045E10Rik*	RIKEN cDNA B530045E10 gene	-10	-4.2
57	*Fras1*	Fraser extracellular matrix complex subunit 1	-10	-3.0
58	*Gm12159*	predicted gene 12159	-10	-2.4
59	*Gm48488*	predicted gene, 48488	-10	-2.5
60	*9030404E10Rik*	RIKEN cDNA 9030404E10 gene	-10	-12
61	*Hmcn1*	hemicentin 1	-10	-3.0
62	*Igkv4-68*	immunoglobulin kappa variable 4-68	-10	-15
63	*Wnt7a*	wingless-type MMTV integration site family, member 7A	-10	-15
64	*Tmem74*	transmembrane protein 74	-9.9	-5.9
65	*Ighv1-66*	immunoglobulin heavy variable 1-66	-9.9	-31
66	*Cr2*	complement receptor 2	-9.5	-5.6

67	*Slc5a11*	solute carrier family 5 (sodium/glucose cotransporter), member 11	-9.5	-71
68	*Lrrc17*	leucine rich repeat containing 17	-9.3	-6.6
69	*Slc22a14*	solute carrier family 22 (organic cation transporter), member 14	-9.2	-1.7
70	*Dipk2b*	divergent protein kinase domain 2B	-9.1	-11
71	*Fgf18*	fibroblast growth factor 18	-9.0	-5.6
72	*Ndp*	Norrie disease (pseudoglioma) (human)	-8.9	-4.6
73	*Gm17334*	predicted gene, 17334	-8.9	-5.7
74	*Btnl2*	butyrophilin-like 2	-8.9	-6.7
75	*Gm10432*	predicted gene 10432	-8.9	-3.6
76	*Colq*	collagen-like tail subunit (single strand of homotrimer) of asymmetric acetylcholinesterase	-8.8	-3.2
77	*Gm48662*	predicted gene, 48662	-8.8	-7.2
78	*4833412C05Rik*	RIKEN cDNA 4833412C05 gene	-8.7	-2.6
79	*Gm12576*	predicted gene 12576	-8.7	-5.3
80	*Igkv6-17*	immunoglobulin kappa variable 6-17	-8.5	-8.2
81	*Gm13816*	predicted gene 13816	-8.3	-8.4
82	*Tmem266*	transmembrane protein 266	-8.3	1.2
83	*Ighv5-6*	immunoglobulin heavy variable 5-6	-8.3	-67
84	*Cntn4*	contactin 4	-8.3	-2.0
85	*Rbp7*	retinol binding protein 7, cellular	-8.1	-2.3
86	*Insyn2a*	inhibitory synaptic factor 2A	-8.0	-3.4
87	*Slc5a12*	solute carrier family 5 (sodium/glucose cotransporter), member 12	-7.9	-3.1
88	*Gm9947*	predicted gene 9947	-7.9	-3.3
89	*Igkv12-89*	immunoglobulin kappa chain variable 12-89	-7.8	-7.3
90	*Gm26795*	predicted gene, 26795	-7.8	-2.0
91	*Ddx4*	DEAD box helicase 4	-7.7	-1.5
92	*Dclk3*	doublecortin-like kinase 3	-7.6	-16
93	*Hpcal4*	hippocalcin-like 4	-7.6	-3.3
94	*Ndnf*	neuron-derived neurotrophic factor	-7.5	-8.3
95	*Igkv17-121*	immunoglobulin kappa variable 17-121	-7.5	-10
96	*Slurp1*	secreted Ly6/Plaur domain containing 1	-7.4	-3.3
97	*Ano4*	anoctamin 4	-7.4	-1.7
98	*Ighv1-7*	immunoglobulin heavy variable V1-7	-7.3	-3.5
99	*Rimklb*	ribosomal modification protein rimK-like family member B	-7.2	-4.8
100	*Igkv4-80*	immunoglobulin kappa variable 4-80	-7.2	-10

Suppl. Tab. ST3A. Genes with Inceased Expression in Kidneys of Infected Mice

			Kidneys	
	Gene ID	Gene Name	Saline	NTCI
1	*Epx*	eosinophil peroxidase	118	231
2	*Gm26965*	predicted gene, 26965	100	30
3	*Reg3g*	regenerating islet-derived 3 gamma	83	116
4	*Cela3b*	chymotrypsin-like elastase family, member 3B	70	-1.0
5	*Gm12503*	predicted gene 12503	64	32
6	*Gpbar1*	G protein-coupled bile acid receptor 1	60	266
7	*Dnah6*	dynein, axonemal, heavy chain 6	58	47
8	*Fut2*	fucosyltransferase 2	50	252
9	*Muc4*	mucin 4	49	87
10	*Gm43189*	predicted gene 43189	37	15
11	*Angptl7*	angiopoietin-like 7	37	11
12	*Pcdh8*	protocadherin 8	32	49
13	*Sftpa1*	surfactant associated protein A1	32	21
14	*Mlana*	melan-A	32	9.7
15	*Gm16350*	predicted gene 16350	31	24
16	*Syt2*	synaptotagmin II	30	15
17	*Gm17055*	predicted gene 17055	29	29
18	*Ppy*	pancreatic polypeptide	29	3.5
19	*Ccdc33*	coiled-coil domain containing 33	29	10
20	*Gm33280*	predicted gene, 33280	28	8.9
21	*Gm45643*	predicted gene 45643	27	15
22	*Lrit3*	leucine-rich repeat, immunoglobulin-like and transmembrane domains 3	26	24
23	*Sbk2*	SH3-binding domain kinase family, member 2	26	26
24	*Slc6a19os*	solute carrier family 6 (neurotransmitter transporter), member 19, opposite strand	26	43
25	*Slamf1*	signaling lymphocytic activation molecule family member 1	26	39
26	*Gm5541*	TD and POZ domain containing 4 pseudogene	26	3.8
27	*Fgf21*	fibroblast growth factor 21	25	5.5
28	*Gm49392*	predicted gene, 49392	25	13
29	*Slc6a16*	solute carrier family 6, member 16	24	-1.0
30	*Olr1*	oxidized low density lipoprotein (lectin-like) receptor 1	24	13
31	*Gm35028*	predicted gene, 35028	23	15
32	*Slfn4*	schlafen 4	23	14
33	*Gm20548*	predicted gene 20548	22	9.3
34	*Gm40709*	predicted gene, 40709	22	9.2
35	*Speer4f2*	spermatogenesis associated glutamate (E)-rich protein 4f2	21	19
36	*Gm13363*	predicted gene 13363	21	14
37	*Egr2*	early growth response 2	20	11
38	*Gm44260*	predicted gene, 44260	19	4.3
39	*Marc1*	mitochondrial amidoxime reducing component 1	19	23
40	*Crygn*	crystallin, gamma N	19	4.5
41	*Lpo*	lactoperoxidase	18	24
42	*Gabrp*	gamma-aminobutyric acid (GABA) A receptor, pi	17	15
43	*Bpifc*	BPI fold containing family C	17	5.0
44	*Fgf18*	fibroblast growth factor 18	17	11
45	*Lncenc1*	long non-coding RNA, embryonic stem cells expressed 1	17	9.1
46	*Prss27*	protease, serine 27	17	39
47	*Muc19*	mucin 19	16	6.9
48	*Gm39929*	predicted gene, 39929	16	3.5
49	*Gm2670*	predicted gene 2670	16	6.7
50	*5830487J09Rik*	RIKEN cDNA 5830487J09 gene	15	10.0
51	*Olfr1442*	olfactory receptor family 5 subfamily B member 94	15	21
52	*Krt20*	keratin 20	14	30
53	*Gm19684*	predicted gene, 19684	14	1.9
54	*Gm33424*	predicted gene, 33424	14	55
55	*4732419C18Rik*	RIKEN cDNA 4732419C18 gene	14	30
56	*Gm37621*	predicted gene, 37621	14	13
57	*Creb3l3*	cAMP responsive element binding protein 3-like 3	13	5.9
58	*Gm14319*	predicted gene 14319	13	18
59	*Dnah10*	dynein, axonemal, heavy chain 10	12	12
60	*Areg*	amphiregulin	12	24
61	*Armc12*	armadillo repeat containing 12	12	29
62	*Eif4ebp3*	eukaryotic translation initiation factor 4E binding protein 3	12	20
63	*Gdf6*	growth differentiation factor 6	11	4.8
64	*Gm49037*	predicted gene, 49037	11	11
65	*Gm42793*	predicted gene 42793	11	41
66	*Derl3*	Der1-like domain family, member 3	11	5.5

67	*Nptx2*	neuronal pentraxin 2	11	23
68	*Atp12a*	ATPase, H+/K+ transporting, nongastric, alpha polypeptide	10	6.9
69	*Kcna7*	potassium voltage-gated channel, shaker-related subfamily, member 7	10	3.3
70	*Prg4*	proteoglycan 4 (megakaryocyte stimulating factor, articular superficial zone protein)	9.8	4.7
71	*Ctla4*	cytotoxic T-lymphocyte-associated protein 4	9.8	16
72	*Gm44164*	predicted gene, 44164	9.4	5.7
73	*Mmp7*	matrix metallopeptidase 7	9.2	4.7
74	*Gm44949*	predicted gene 44949	9.2	8.9
75	*Slc17a8*	solute carrier family 17 (sodium-dependent inorganic phosphate cotransporter), member 8	8.9	2.9
76	*Lamb3*	laminin, beta 3	8.7	5.9
77	*Plekhs1*	pleckstrin homology domain containing, family S member 1	8.7	19
78	*Trim17*	tripartite motif-containing 17	8.7	2.8
79	*Dusp8*	dual specificity phosphatase 8	8.7	6.2
80	*4930422C21Rik*	RIKEN cDNA 4930422C21 gene	8.6	3.7
81	*Gm10727*	predicted gene 10727	8.6	2.3
82	*Col27a1*	collagen, type XXVII, alpha 1	8.5	8.5
83	*Tacc2*	transforming, acidic coiled-coil containing protein 2	8.3	5.9
84	*Fat2*	FAT atypical cadherin 2	8.3	2.4
85	*Lif*	leukemia inhibitory factor	8.1	20
86	*Klk1b1*	kallikrein 1-related peptidase b1	8.0	4.6
87	*Tsc22d1*	TSC22 domain family, member 1	7.9	11
88	*Wnt8b*	wingless-type MMTV integration site family, member 8B	7.8	20
89	*Grem1*	gremlin 1, DAN family BMP antagonist	7.7	14
90	*Hesx1*	homeobox gene expressed in ES cells	7.7	15
91	*Hhipl1*	hedgehog interacting protein-like 1	7.7	5.3
92	*Ugt1a5*	UDP glucuronosyltransferase 1 family, polypeptide A5	7.6	1.3
93	*Klk1b4*	kallikrein 1-related pepidase b4	7.5	15
94	*Gm8947*	predicted gene 8947	7.4	6.0
95	*A530013C23Rik*	RIKEN cDNA A530013C23 gene	7.4	9.5
96	*Mylk4*	myosin light chain kinase family, member 4	7.3	3.6
97	*Cyp2a5*	cytochrome P450, family 2, subfamily a, polypeptide 5	7.2	9.0
98	*Muc1*	mucin 1, transmembrane	6.9	4.5
99	*Gm44588*	predicted gene 44588	6.9	2.6
100	*Gm45053*	predicted gene 45053	6.8	10

Suppl. Tab. ST3B. Genes with Decreased Expression in Kidneys of Infected Mice

			Kidneys	
	Gene ID	Gene Name	Saline	NTCI
1	*Cyp11b1*	cytochrome P450, family 11, subfamily b, polypeptide 1	-70,748	-1,178
2	*Cyp11a1*	cytochrome P450, family 11, subfamily a, polypeptide 1	-27,744	-483
3	*Akr1cl*	aldo-keto reductase family 1, member C-like	-17,566	-884
4	*Hsd3b1*	hydroxy-delta-5-steroid dehydrogenase, 3 beta- and steroid delta-isomerase 1	-11,966	-476
5	*Scg2*	secretogranin II	-4,862	-674
6	*Th*	tyrosine hydroxylase	-4,501	-344
7	*Cyp21a1*	cytochrome P450, family 21, subfamily a, polypeptide 1	-3,966	-495
8	*Chga*	chromogranin A	-3,318	-793
9	*Cyp21a2-ps*	cytochrome P450, family 21, subfamily a, polypeptide 2 pseudogene	-1,979	-443
10	*Pnmt*	phenylethanolamine-N-methyltransferase	-1,705	-322
11	*Chgb*	chromogranin B	-1,447	-508
12	*Cyp11b2*	cytochrome P450, family 11, subfamily b, polypeptide 2	-1,274	-114
13	*Caln1*	calneuron 1	-1,121	NS
14	*Kcnk9*	potassium channel, subfamily K, member 9	-1,062	-238
15	*Dbh*	dopamine beta hydroxylase	-912	-641
16	*Gm28382*	predicted gene 28382	-869	-348
17	*Pla2g2f*	phospholipase A2, group IIF	-851	NS
18	*Nr5a1*	nuclear receptor subfamily 5, group A, member 1	-843	-988
19	*Pcsk1n*	proprotein convertase subtilisin/kexin type 1 inhibitor	-707	-97
20	*Mgarp*	mitochondria localized glutamic acid rich protein	-666	-244
21	*4930486L24Rik*	RIKEN cDNA 4930486L24 gene	-630	-23
22	*Cryba4*	crystallin, beta A4	-549	-43
23	*Atp2b3*	ATPase, Ca++ transporting, plasma membrane 3	-541	NS
24	*Resp18*	regulated endocrine-specific protein 18	-510	-332
25	*E330017L17Rik*	RIKEN cDNA E330017L17 gene	-473	-154
26	*Pcsk1*	proprotein convertase subtilisin/kexin type 1	-468	-57
27	*Snap25*	synaptosomal-associated protein 25	-448	NS
28	*Rims4*	regulating synaptic membrane exocytosis 4	-352	-271
29	*Slc24a2*	solute carrier family 24 (sodium/potassium/calcium exchanger), member 2	-344	NS
30	*Phox2b*	paired-like homeobox 2b	-316	NS
31	*Slc18a2*	solute carrier family 18 (vesicular monoamine), member 2	-286	-31
32	*Eef1a2*	eukaryotic translation elongation factor 1 alpha 2	-271	-181
33	*Chrna3*	cholinergic receptor, nicotinic, alpha polypeptide 3	-260	-169
34	*Hand2os1*	Hand2, opposite strand 1	-240	NS
35	*Cntnap5a*	contactin associated protein-like 5A	-240	-38
36	*Rasgrf1*	RAS protein-specific guanine nucleotide-releasing factor 1	-236	-26
37	*Hand2*	heart and neural crest derivatives expressed 2	-226	-951
38	*Pnmal1*	PNMA family member 8A	-221	-144
39	*Necab2*	N-terminal EF-hand calcium binding protein 2	-184	-44
40	*Tbx20*	T-box 20	-182	NS
41	*Stmn3*	stathmin-like 3	-163	-22
42	*Gm17322*	predicted gene, 17322	-154	-17
43	*St8sia3*	ST8 alpha-N-acetyl-neuraminide alpha-2,8-sialyltransferase 3	-148	NS
44	*Rab33a*	RAB33A, member RAS oncogene family	-145	-97
45	*Synpr*	synaptoporin	-140	-11
46	*Rit2*	Ras-like without CAAX 2	-110	-11
47	*Camkv*	CaM kinase-like vesicle-associated	-107	NS
48	*Gdap1*	ganglioside-induced differentiation-associated-protein 1	-105	NS
49	*Soga3*	SOGA family member 3	-99	NS
50	*Mdga2*	MAM domain containing glycosylphosphatidylinositol anchor 2	-99	-10
51	*Mab21l2*	mab-21-like 2	-96	-122
52	*Nrsn1*	neurensin 1	-93	-61
53	*Cadps*	Ca2+-dependent secretion activator	-91	-37
54	*Gnb3*	guanine nucleotide binding protein (G protein), beta 3	-83	NS
55	*1520401A03Rik*	glycine rich extracellular protein 1	-82	NS
56	*Nrg3*	neuregulin 3	-82	NS
57	*Spock1*	sparc/osteonectin, cwcv and kazal-like domains proteoglycan 1	-81	-8.7
58	*Cdk5r2*	cyclin dependent kinase 5, regulatory subunit 2 (p39)	-78	NS
59	*Ina*	internexin neuronal intermediate filament protein, alpha	-76	-26
60	*Actl6b*	actin-like 6B	-75	NS
61	*Npy2r*	neuropeptide Y receptor Y2	-74	-21
62	*Gal*	galanin and GMAP prepropeptide	-70	-49
63	*Nrsn2*	neurensin 2	-69	NS
64	*Gabrg2*	gamma-aminobutyric acid (GABA) A receptor, subunit gamma 2	-69	NS
65	*Gpr158*	G protein-coupled receptor 158	-68	NS
66	*Slc7a14*	solute carrier family 7 (cationic amino acid transporter, y+ system), member 14	-68	-32

67	*Sphkap*	SPHK1 interactor, AKAP domain containing	-67	-51
68	*Tmem255a*	transmembrane protein 255A	-67	-61
69	*8430419K02Rik*	RIKEN cDNA 8430419K02 gene	-66	-2.9
70	*Scg3*	secretogranin III	-65	-276
71	*Gria1*	glutamate receptor, ionotropic, AMPA1 (alpha 1)	-65	-11
72	*Txndc2*	thioredoxin domain containing 2 (spermatozoa)	-63	-59
73	*Gm27477*	predicted gene, 27477	-63	-2.9
74	*Gria2*	glutamate receptor, ionotropic, AMPA2 (alpha 2)	-62	-154
75	*Gjd2*	gap junction protein, delta 2	-62	NS
76	*Lrrtm2*	leucine rich repeat transmembrane neuronal 2	-62	-2.0
77	*Hsd3b6*	hydroxy-delta-5-steroid dehydrogenase, 3 beta- and steroid delta-isomerase 6	-62	-52
78	*Diras1*	DIRAS family, GTP-binding RAS-like 1	-62	-40
79	*Omg*	oligodendrocyte myelin glycoprotein	-61	NS
80	*Gm49391*	predicted gene, 49391	-60	-6.3
81	*Fam155a*	NALCN channel auxiliary factor 1	-59	-146
82	*Gm2007*	predicted gene 2007	-59	-4.6
83	*Kcnq2*	potassium voltage-gated channel, subfamily Q, member 2	-58	-38
84	*Gm42372*	PNMA family member 8C	-57	-5.8
85	*Isl1*	ISL1 transcription factor, LIM/homeodomain	-57	-7.2
86	*Lingo3*	leucine rich repeat and Ig domain containing 3	-57	-20
87	*Prl8a2*	prolactin family 8, subfamily a, member 2	-57	NS
88	*Adh7*	alcohol dehydrogenase 7 (class IV), mu or sigma polypeptide	-56	-20
89	*Lgi1*	leucine-rich repeat LGI family, member 1	-53	-7.1
90	*Gm47815*	predicted gene, 47815	-53	NS
91	*Gm14592*	splicing factor 3a, subunit 3 pseudogene	-52	NS
92	*Cdx4*	caudal type homeobox 4	-52	-21
93	*Marcksl1-ps4*	MARCKS-like 1, pseudogene 4	-52	-3.1
94	*Sstr3*	somatostatin receptor 3	-48	-3.1
95	*Pcdh10*	protocadherin 10	-47	-8.6
96	*Gm27000*	predicted gene, 27000	-47	NS
97	*Tub*	tubby bipartite transcription factor	-47	-20
98	*Mc2r*	melanocortin 2 receptor	-46	-53
99	*Il20ra*	interleukin 20 receptor, alpha	-44	-7.0
100	*Grin2a*	glutamate receptor, ionotropic, NMDA2A (epsilon 1)	-43	-28
